# Supplementary material for: Mutation rates and fitness consequences of mosaic chromosomal alterations in blood
Source: Nat Genet. 2023 Sep 11;55(10):1677–85. doi: 10.1038/s41588-023-01490-z (PMC10562253; doi:10.1038/s41588-023-01490-z)
Supplement: Supplementary file 1 — Supplementary Notes 1–5 and Supplementary Figs. 1–27. [file 41588_2023_1490_MOESM1_ESM.pdf]

nature genetics

Article https://doi.org/10.1038/s41588-023-01490-z

# Mutation rates and fitness consequences of mosaic chromosomal alterations in blood

In the format provided by the authors and unedited

# Supplementary Notes


**1 Effect of mCA detection limits on fitness and mutation rate estimates 3**

**2 Age dependence of mCAs 4**

**3 Length dependence of loss events involving specific genes 5**

**4 Relationship between mutation rate or fitness effect and more descriptive metrics 5**

**5 Distribution of cell fractions for high mutation rate events 5**


# Supplementary Figures


Supplementary Figure 1 mCAs detected among ∼500,000 UK Biobank participants: part 1 . . . 7
Supplementary Figure 2 mCAs detected among ∼500,000 UK Biobank participants: part 2 . . . 8
Supplementary Figure 3 Number of observations of each mCA in Loh 2020, in people who had a total of 1, 2 or >2 mCAs detected. . . . 9
Supplementary Figure 4 Number of autosomal mCAs per person . . . 10
Supplementary Figure 5 Effect of upper and lower mCA detection limits on fitness inferences . . . 11
Supplementary Figure 6 Effect of upper and lower mCA detection limits on mutation rate inferences . . . 12
Supplementary Figure 7 Effect of false negatives at low cell fraction on fitness and mutation rate inferences . . . 13
Supplementary Figure 8 Sex differences in fitness effects and mutation rates: gains . . . 14
Supplementary Figure 9 Sex differences in fitness effects and mutation rates: losses: part 1 . . . 15
Supplementary Figure 10 Sex differences in fitness effects and mutation rates: losses: part 2 . . . 16
Supplementary Figure 11 Sex differences in fitness effects and mutation rates: CN-LOH: part 1 . . . 17
Supplementary Figure 12 Sex differences in fitness effects and mutation rates: CN-LOH: part 2 . . . 18
Supplementary Figure 13 Sex differences in fitness effects and mutation rates: CN-LOH: part 3 . . . 19
Supplementary Figure 14 Sex differences in fitness effects and mutation rates: CN-LOH: part 4 . . . 20
Supplementary Figure 15 Age and sex dependence of individual mCAs: part 1 . . . 21
Supplementary Figure 16 Age and sex dependence of individual mCAs: part 2 . . . 22
Supplementary Figure 17 Age and sex dependence of individual mCAs: part 2 . . . 23
Supplementary Figure 18 Quantifying deviation from age dependence . . . 24
Supplementary Figure 19 Deviation from expected age-dependence for mCAs with poor age dependence . . . 25
Supplementary Figure 20 Examining mCAs with poor age dependence: part 1 . . . 26
Supplementary Figure 21 Examining mCAs with poor age dependence: part 2 . . . 27
Supplementary Figure 22 Examining mCAs with poor age dependence: part 3 . . . 28
Supplementary Figure 23 Age dependence of the distribution of clone sizes for specific mCAs . . . 29
Supplementary Figure 24 Fitness effects and mutation rates for mCAs observed as single events vs. observed with additional mCAs . . . 30
Supplementary Figure 25 Length dependence of fitness effects and mutation rates for loss events . . . 31
Supplementary Figure 26 Relationship between mCA mutation rate or fitness effect and more descriptive metrics . . . 32
Supplementary Figure 27 Distribution of simulated mLOY and mLOX cell fractions, with high mutation rates . . . 33

# 1. Effect of mCA detection limits on fitness and mutation rate estimates

**mCA cell fraction detection limits.**

The upper cell fraction limit of detection, for the mCA calls generated by Loh et al [1], was 67% for losses and 54% for CN-LOH events, corresponding to BAF deviations >0.25. This was due to the analytical approach used for calling, which resulted in heterozygous SNPs 'dropping out' of the data if BAF deviations were >0.25. The lower cell fraction limit of detection depends on both the mCA length (with longer events being easier to detect) and the mCA type, with CN-LOH producing greater BAF deviations than loss and gain events of the same cell fraction. In order to provide a conservative estimate for the lower limit of detection for an mCA of a given class, e.g. CN-LOH, we recorded the lowest cell fraction detected for each mCA in the class, multiplied this by 1.5 (to reduce the false negative rate) and took the maximum of these values. Because we are taking the maximum of the lower thresholds, we expect that the false negative rate for most chromosomes will be low. Our inferred lower cell fraction limits of detection were 2.5% for gains, 4.1% for losses and 1.5% for CN-LOH events (Supplementary Table 7).

**Simulated data to determine effect of mCA detection limits.**

In order to assess how cell fraction detection limits impact our estimates of fitness effects and mutation rates, we performed extensive simulations of the process. Our framework is able to accurately recover the ground-truth values for both fitness and mutation rate across a wide-range of parameter values. This range is slightly reduced because of cell fraction detection limits (outlined below), but these simulations show that we expect the vast majority of our inferences to be accurate.

We generated simulated mCA calls using a custom written stochastic process implemented in Python (version 3.7.4). Briefly:

- The code simulates a population (`population`), which is a list of all unique clones, and their abundances, present in the population at a given time.
- Clones are tuples composed of `(clone_ID, clone_size)` where `clone_size` is the integer number of cells comprising the clone, and, where `clone_ID` is a list of `(mutation_ID, fitness_effect)` pairs for all unique mutations that have accumulated in that clone.
- `mutation_ID` uniquely labels each independent mutation to have entered the population and is updated via a counter called `last_mutation_ID`, thus a `mutation_ID=5` means that was the 5th mutation to occur in the population.
- `fitness_effect` is the selective effect, $s$, of the mutation and is defined at the start of the simulation. All mutated clones generated by the simulation will have the same defined fitness effect.
- The dynamics are implemented using two functions: `mutate` (which generate new clones) and `divide` (which modifies the clone sizes of existing clones) in discrete time steps, where units of time are measured in years.
- The function `mutate` creates new clones by querying each clone in the current population and determining the number of daughter clones each gives rise to by drawing a Poisson random variate with mean `clone_size` × $\mu$ × `dt`, where $\mu = 4.35 \times 10^{-9}$ is the mutation rate for each cell in the clone. New clones are added to the list of all clones with an initial `clone_size = 1`. In this simple simulation, further mutations cannot occur if the clone already carries a mutation.
- The function `divide` updates the clone sizes of existing clones determining the difference in the number of cell-births and cell-deaths occurring in that clone. In the time interval `dt` these are calculated via

  $$\texttt{births} = X(\lambda \times \texttt{B} \times \texttt{clone\_size} \times \texttt{dt}) \quad \textbf{(5)}$$

  where $X(g)$ is a Poisson random variate with mean $g$ and where $\lambda = 5$ is the total cell division rate (i.e. we assume 5 HSC divisions per year). Similarly the number of deaths is

  $$\texttt{deaths} = X(\lambda \times \texttt{D} \times \texttt{clone\_size} \times \texttt{dt}) \quad \textbf{(6)}$$

  The fitness differences are implemented by allowing birth-rates to depend on the fitness of the mutations in the clone:

  $$\texttt{B} = \texttt{B0} \times (1 + F) \quad \textbf{(7)}$$

  where $\texttt{D} = \texttt{B0} = 0.2$. Thus, when there is no fitness advantage ($F = 0$) the clone performs neutral drift due to matched birth and death rates i.e. one in every 5 cell divisions results in a self-renewal, and another 1 in 5 results in a symmetric differentiation event. The total fitness advantage ($F$) is determined by summing the fitness effects of all the mutations the clone has acquired ($F = \sum_i s_i$). In this simple simulation, where further mutations cannot occur if a clone already carries a mutation, $F$ is simply equal to the mutation fitness effect defined at the start of the simulation.
- The `clone_size` and `population_size` are then recorded at any time (age) required and are used to calculate the cell fraction, `cell_fraction`, where `cell_fraction` = `clone_size`/`population_size`.

For each integer fitness effect in the range 7% to 35% per year, we generated simulated mCA calls across ∼500,000 individuals, using the actual ages from UK Biobank. At fitness effects <7%, there were not enough mCA calls across the ∼500,000 individuals to generate a meaningful cell fraction density histogram to infer the fitness effects and mutation rates. The number of simulated mCAs 'observed' (across ∼500,000 simulated individuals) according to the fitness effect of the mCA and the cell fraction limits of detection are shown in Supplementary Table 8.

### Effect of mCA detection limits on fitness and mutation rate inferences.

If the exponential fall-off in cell fraction ($\phi$ in eq. 1) occurs at a higher cell fraction than the upper cell fraction limit of detection for the mCA, this might be expected to impair our ability to accurately infer the fitness effect of that mCA. Similarly, if $\phi$ occurs at a lower cell fraction than the lower cell fraction limit of detection for the mCA, this might be expected to impair our ability to infer both the fitness effect and the mutation rate of the mCA. To test this, we used our simulated mCA calls to assess the effect of the lower and upper cell fraction limit of detection on i) our fitness effect inferences (Supplementary Fig. 5), and ii) our mutation rate inferences (Supplementary Fig. 6). Fitness effects and mutation rates were inferred as described in Methods.

***Fitness effect inferences.***

When there is no upper cell fraction limit of detection (Supplementary Fig. 5a) (as is the case for gains), we are able to reliably infer the fitness effect of mCAs to at least the maximum simulated fitness effect (35% per year), although the upper error bars become large for fitness effects $\geq$26% per year. When there is an upper cell fraction limit of detection this affects our ability to infer the fitness effect of high fitness mCAs (Supplementary Fig. 5b). For CN-LOH events, which have an upper cell fraction limit of detection of 54%, we can reliably infer the fitness effect up to ∼25% per year. For loss events, which have an upper cell fraction limit of detection of 67% per year, we can reliably infer the fitness effect up to ∼24% per year (Supplementary Table 9).

A lower cell fraction limit of detection affects our ability to infer the fitness effect of low fitness mCAs (Supplementary Fig. 5a). For CN-LOH and gain events, which have a lower cell fraction limits of detection of 1.5% and 2.5% respectively, we can infer the fitness effect down to 7% per year. For loss events, which have a lower cell fraction limit of detection of 4.1%, we can infer the fitness effect down to 9% per year (Supplementary Table 9).

***Mutation rate inferences.***

Whether or not there is an upper cell fraction limit of detection makes little difference to our ability to reliably infer mutation rates (Supplementary Fig. 6a-b), because our framework uses the amplitude of cell fraction densities at low cell fractions to infer mutation rates. The lower cell fraction limit of detection and the fitness effect of the mCA affects how many mCAs we see at low cell fractions, which is reflected by the slight decrease in accuracy of our mutation rate inferences at fitness effects $\leq$10% per year (Supplementary Fig. 6a-b). Nonetheless, for our lowest fitness mCAs (Fig. 2, CN-LOH at fitness effects of $\leq$9% per year), our mutation rate inferences are still accurate to within a factor of 2.

### Effect of false negatives at low cell fraction on fitness and mutation rate inferences.

Smaller mCAs are harder to detect and so these events could potentially have a higher false negative rates in the data. A key strength of our framework is that it is largely insensitive to these issues. By fitting the predicted distribution (governed by mutation rate and fitness effect), our framework integrates joint information about how many mCA events there are in each cell fraction interval. This means our estimates are not dominated by the large number of events at low cell fractions where false negative rates would have a large effect on estimates.

To demonstrate that our method is robust to false negatives at low cell fraction, we reanalysed all the (non-simulated) mCA data, but only included mCAs whose cell fractions were high enough that they were not substantially affected by false negatives (Supplementary Fig. 7). This shows that our inferences are robust even in the presence of false negatives at low cell fraction (Supplementary Fig. 7d-e).

## 2. Age dependence of mCAs

### Quantifying deviation from expected age dependence.

To quantify any deviation from the expected age dependence, we calculated the relative difference between the gradients of the observed and expected age dependence (Supplementary Fig. 18). A maximum likelihood approach was used to calculate the gradients ($m_{\text{obs}}$ or $m_{\text{exp}}$), minimising the L2 norm between the observed or expected prevalence ($P$) and $y = mt + C$ across 3 age ($t$) groups (age 40-50, 50-60, 60-70), optimising $m$ and $C$. To account for error in prevalence, the square distance was multiplied by $T/2P$, where $T$ is the total number of people in the age group. An age group was excluded from both observed and expected gradient estimation if the observed prevalence in that age group was 0 (because the prevalence would not be expected to be increasing linearly at that age). To determine whether any deviation from expected age dependence was statistically significant, a

distribution of difference probability curve was calculated (for observed vs. expected age dependence gradients) and the *p*-value of the difference was calculated as the area under the curve where the difference ≤0. If the *p*-value was <0.05, the deviation from expected age dependence was deemed statistically significant. The relative difference, $(m_{\text{obs}}\text{-}m_{\text{exp}})/m_{\text{exp}}$, between the observed and expected age dependence gradients for individual mCAs is shown in Fig. 3d and the *p*-values in Supplementary Table 3.

**Can decline in prevalence with age for some mCAs be explained by acquisition of additional mCAs?.**

For the mCAs that weaker age dependence than expected (i.e. less increase in prevalence with age than expected), we reasoned that could this be because individuals with these mCAs are more likely to acquire additional mCAs with increasing age, which effectively lowers the prevalence of 'single mCAs' in older age groups. To check this, we looked at the age prevalence for the mCAs that showed significantly weaker age dependence than expected and checked whether this age dependence improved by including individuals that had ≥1 mCA (relative to its expected prevalence based on our model). For 18/33 mCAs, the age dependence remained significantly worse than expected (Supplementary Fig. 19 and Supplementary Figs. 20-22), suggesting the poor age dependence observed in these mCAs is not due to the acquisition of additional mCAs. For 15/33 mCAs the age dependence was no longer significantly worse than expected (Supplementary Fig. 19 and Supplementary Figs. 20-22), suggesting the poor age dependence seen in some mCAs may be due to the acquisition of additional mCAs; this was the case for 2/2 gains (21q+ in women, 22q+ in men), 3/4 losses (2p- in women, 5q- in women, 11q- in men) and 10/27 CN-LOH events.

## 3. Length dependence of loss events involving specific genes

Strong clustering of loss events can be seen involving genes recurrently mutated in clonal haematopoiesis and haematological malignancies, e.g. *DNMT3A*, *TET2*, *DLEU1*, *IGH* (Supplementary Fig. 25a), suggesting the fitness effect conferred by these loss events might be attributable to the loss of one of the cell's copies of these genes. We wondered whether the fitness effects of these loss events were similar to the fitness effects inferred for SNVs in these genes[2] and how the fitness effects and mutation rates depended on the length of the chromosomal section lost. To assess this, loss events involving these genes were separated in to length categories (0-3 MB, 3-10 MB, 10-30 MB and 30-100 MB) and the fitness effects and mutations rates for the loss events within each length category were inferred using our evolutionary framework (as described in Methods) (Supplementary Fig. 25b, c, Supplementary Table 6).

Some confidence intervals were large, due to small numbers of events in some length categories (≥ 5 events required), but for the majority of loss events the fitness effect seemed to be unaffected by the length of the loss, suggesting loss of the recurrently mutated gene was the main driver of the fitness effect (Supplementary Fig. 25b). In further support of this, the fitness effects of losses involving *DNMT3A*, *TET2* and *ASXL1* were broadly consistent with the fitness estimates we had previously inferred for SNVs in these genes[2]. The fitness effects of loss events on chromosome 20, involving *ASXL1* and/or *L3MBTL1*, appeared to decrease for loss lengths >30 MB, suggesting the additional loss of a gene (or region) at the telomeric end of chromosome 20 might be having a negative effect on the fitness effect. There was not a consistent pattern for how the mutation rate varied for different lengths of loss involving these genes. With increasing length of loss, the mutation rate seemed to decrease for some genes (e.g. *DNMT3A*, *DLEU2*), but seemed to increase for others, e.g. *ASXL1*.

## 4. Relationship between mutation rate or fitness effect and more descriptive metrics

Descriptive metrics, such as a variant's prevalence, or the proportion observed at higher cell fractions are sometimes used as a proxy for a variant's mutation rate and fitness effect respectively. However, the prevalence of a particular variant (and how that prevalence changes with age) is shaped by *both* the variant's mutation rate and its fitness effect. Furthermore, prevalence is strongly influenced by the sensitivity of the assay and false negative rates at low cell fractions. A strength of our framework is that it is not sensitive to these issues (Supplementary Note 1). Reporting prevalence instead of $\mu$ is problematic because (i) it is not easily comparable across studies which have different sensitivities and across different variants with varying detectability (ii) it does not elucidate the cause of that prevalence (i.e. higher mutation rate vs. higher fitness). Reporting a metric of "fraction of events with a cell fraction above e.g. 5%" rather that $s$ is also problematic as this is confounded by mutation rate and requires arbitrary cell fraction thresholds. The relationship between mCA observations vs. mutation rate and mCA mutation rate vs. proportion at >5% cell fraction are shown in Supplementary Fig. 26.

## 5. Distribution of cell fractions for high mutation rate events

A key difference between allosomal and autosomal mCAs is that we infer both mLOY and mLOX to occur at a high mutation rate, such that the product $\theta = N\tau\mu$ may no longer be a very small parameter ($\theta \ll 1$). In this regime it is likely that multiple independent allosomal mCA events expand at the same time within the same individual. Because of the way in which mCA cell fractions are called, this results in mLOY cell fraction being recorded as the sum of the independent cell fractions in that individual. For mLOX, the cell fraction recorded would be the absolute difference between the sum of the independent

mLOX events affecting the maternal X-chromosome and the sum of the independent mLOY events affecting the paternal X-chromosome. To test whether these effects could result in the qualitative features observed in the mLOY and mLOX distributions in the UK Biobank data we simulated mCA calls at 3 different mutation rates ($\mu = 1 \times 10^{-6}$, $\mu = 1 \times 10^{-5}$ and $\mu = 1 \times 10^{-4}$), corresponding to $\theta = 0.1$, $\theta = 1$ and $\theta = 10$. We compared the simulated distribution of mCA cell fractions to the analytical expression (eq. 8) for the distribution of the sum of exponentially growing clones fed at a constant rate[3].

$$\rho(l) = \frac{1}{\Gamma(\theta)} \times \frac{e^{-\frac{e^l}{\phi}/(1-e^l)}}{\left(\frac{e^l}{\phi}/(1-e^l)\right)^{1-\theta}} \times \frac{e^l}{(1-e^l)^2} \times \frac{1}{\phi} \qquad \text{where } \theta = N\tau\mu \qquad \text{and } \phi = \frac{e^{st}-1}{N\tau s} \tag{8}$$

We considered whether the features in the distributions we observed in Fig. 5 may result from high mutation rate to mLOY and mLOX and the inability to distinguish independent events from the sum (mLOY) or the difference (mLOX). However, as can be seen in Supplementary Fig. 27, these effects produce features that are inconsistent with the features we observed in the real data, suggesting that these effects are not contributing. Multiple independent indistinguishable clones contributing significantly to the sum is also inconsistent with the mutation rates we infer for mLOY and mLOX where we estimate that the value of $\theta \sim 0.1$. In this parameter regime we do not expect these effects to play a significant role in shaping the distribution, as can be seen the the first column of Supplementary Fig. 27.

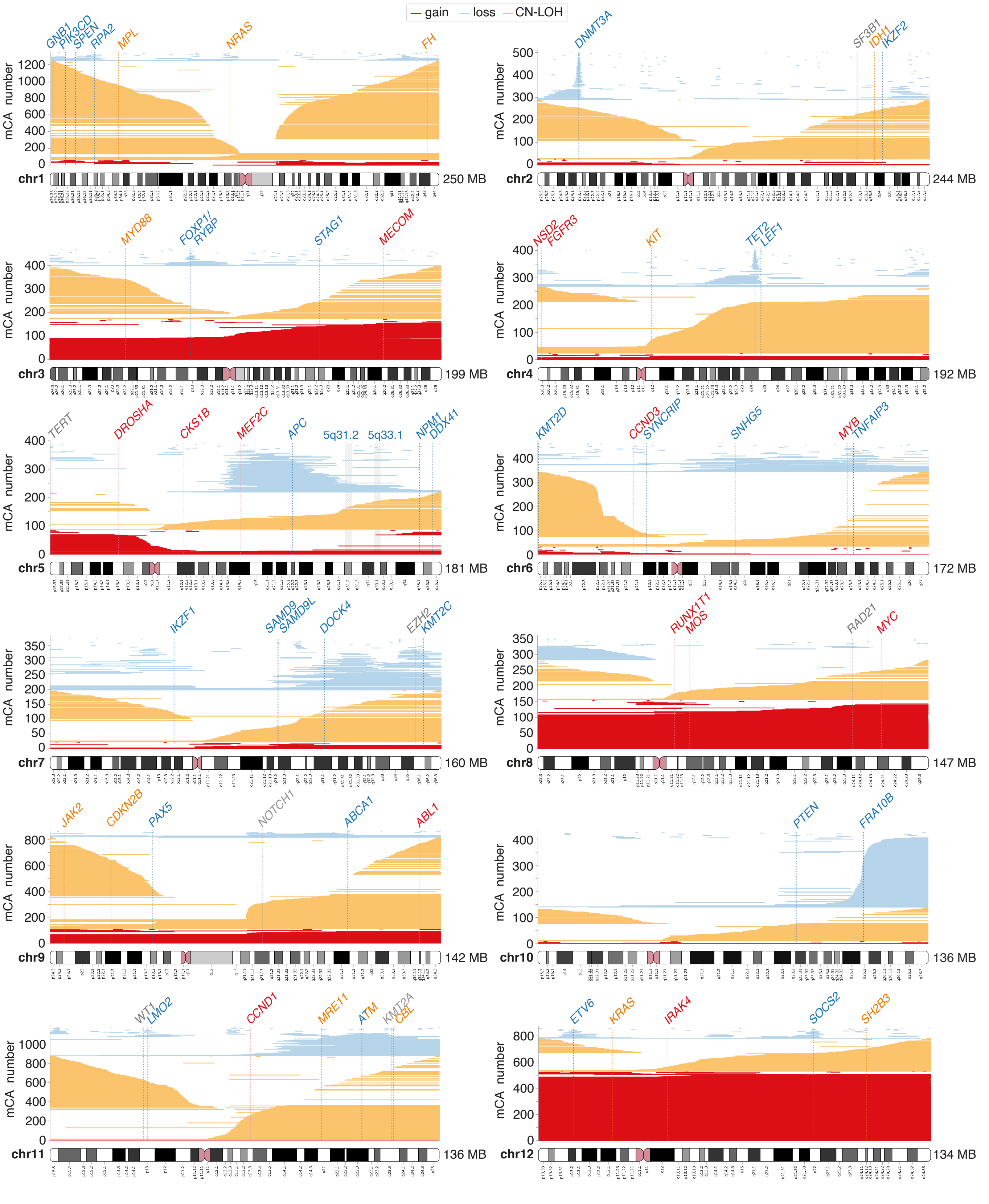


**Supplementary Fig. 1. mCAs detected among ∼500,000 UK Biobank participants in Loh et al 2020 [1]: part 1.** Each mCA is represented as a horizontal line. Gain events are shown in red, loss events in blue and CN-LOH events in yellow. Genes recurrently mutated in clonal haematopoiesis or haematological malignancies which may be putative target genes for loss, gain or CN-LOH events are labelled in blue, red and orange respectively.

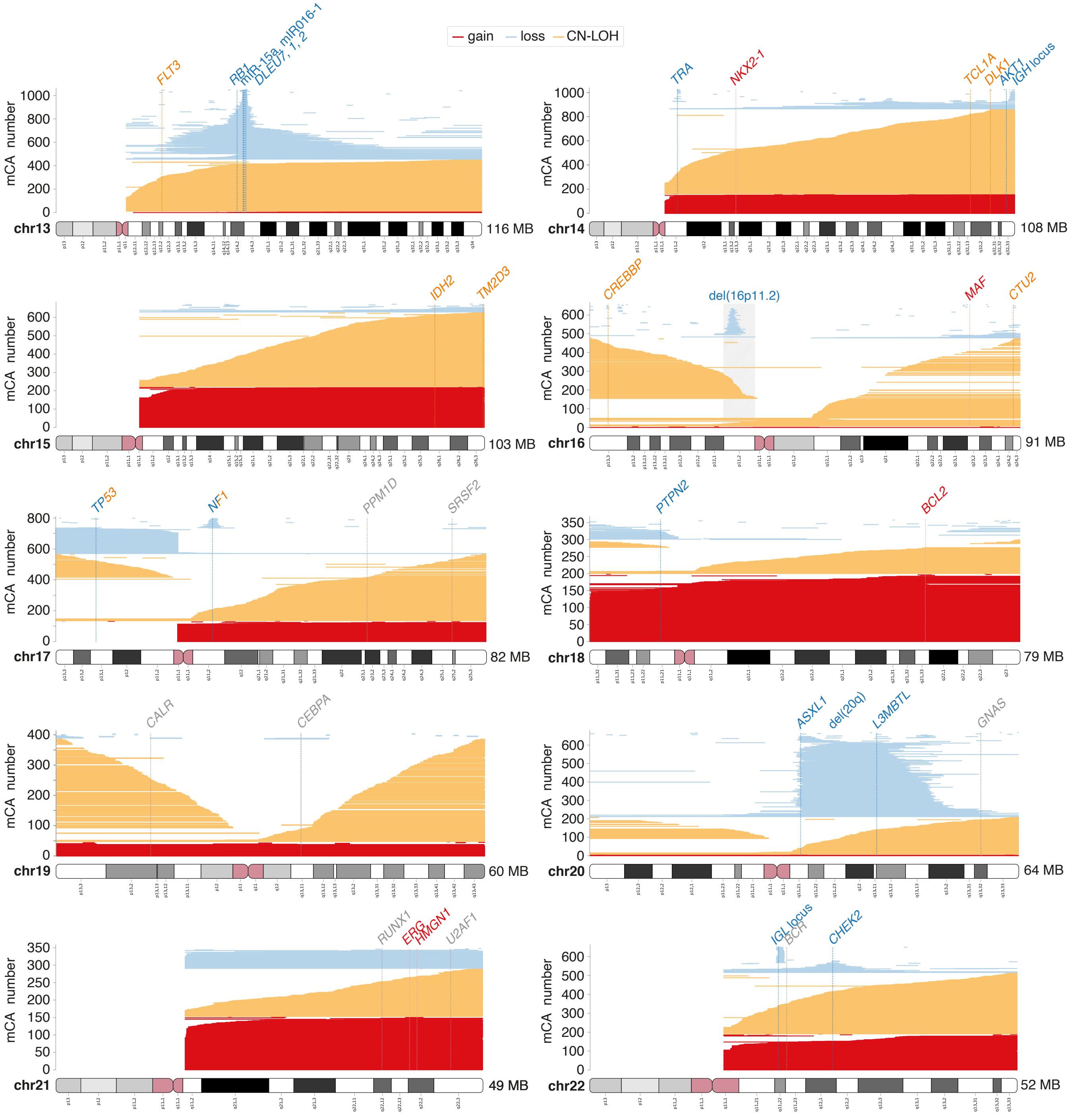


**Supplementary Fig. 2. mCAs detected among $\sim$ 500,000 UK Biobank participants in Loh et al 2020[1]: part 2.** Each mCA is represented as a horizontal line. Gain events are shown in red, loss events in blue and CN-LOH events in yellow. Genes recurrently mutated in clonal haematopoiesis or haematological malignancies which may be putative target genes for loss, gain or CN-LOH events are labelled in blue, red and orange respectively.

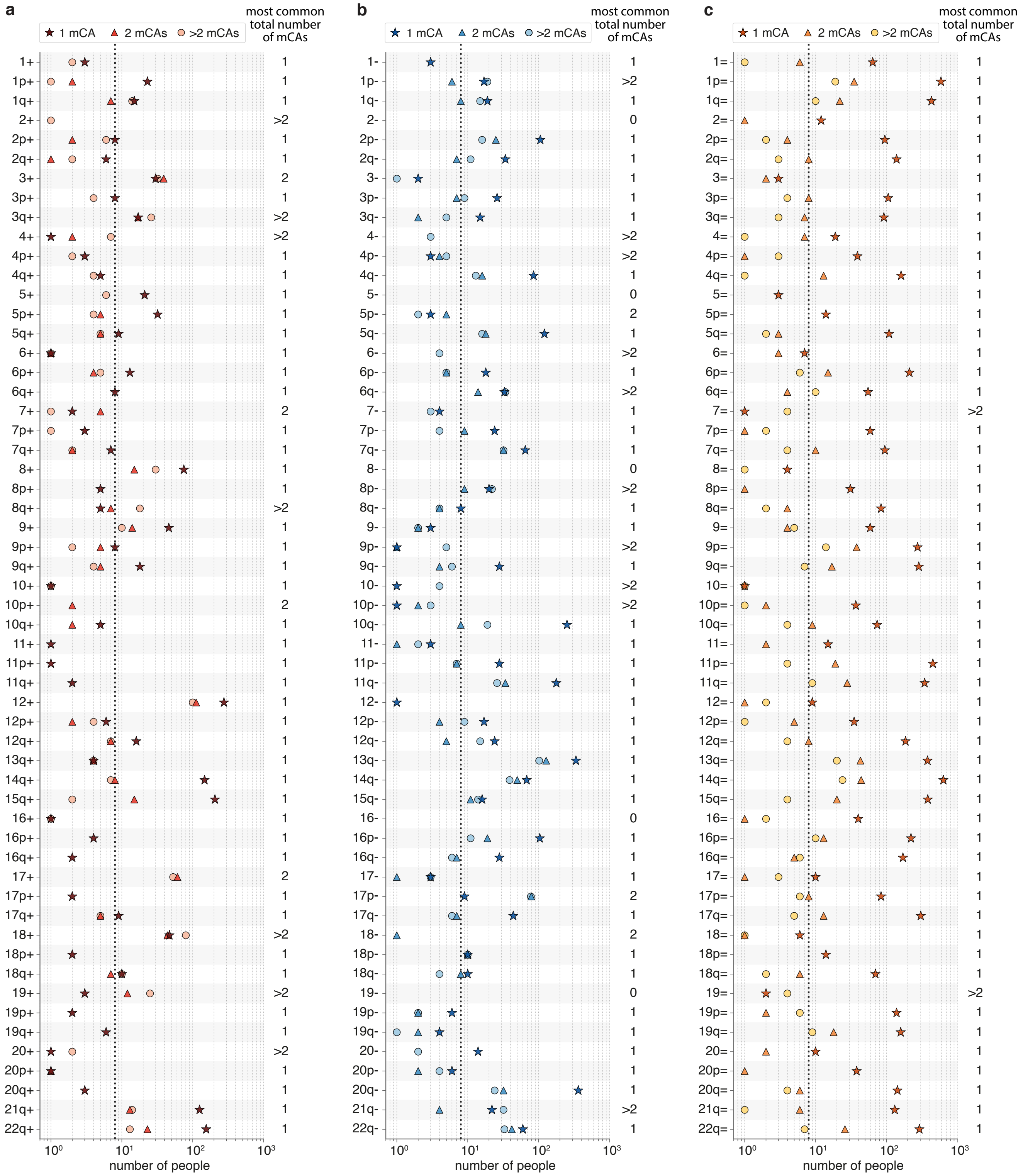


**Supplementary Fig. 3. Number of observations of each mCA in Loh 2020 [1], in people who had a total of 1, 2 or >2 mCAs detected. a.** Gain mCAs. **b.** Loss mCAs. **c.** CN-LOH mCAs. The dashed vertical line indicates the minimum number of people (8) in whom an mCA had to be observed in order to calculate the mCA's fitness effect and mutation rate. The majority of mCAs were most commonly seen in individuals as single events ('most common total number of mCAs: 1'). mCAs that were seen more often in people that had 1 other additional mCA were 3+, 7+, 10p+, 17+, 5p-, 17p-, 18-. mCAs that were seen more often in people that had 2 or more additional mCAs were 2+, 3q+, 4+, 8q+, 18+, 19+, 20+, 1p-, 4-, 4p-, 6-, 6q-, 8p-, 9p-, 10-, 10p-, 21q-, 7=, 19=. 6 mCAs were never seen as single events : 2+, 17+, 4-, 6-, 18-. 5 mCAs were not observed at all: 2-, 5-, 8-, 16-, 19-.

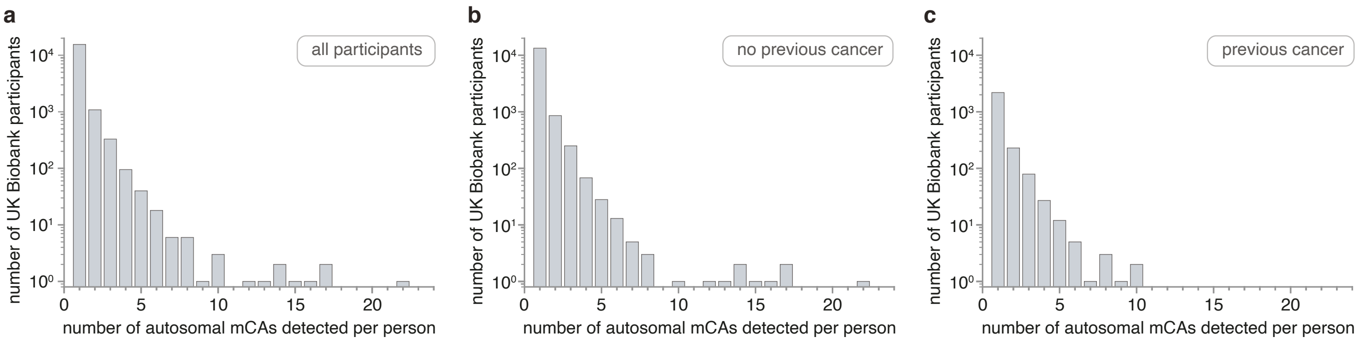


**Supplementary Fig. 4. Number of autosomal mCAs per person, for individuals with an autosomal mCA detected. a.** All individuals with an autosomal mCA detected (mean number mCAs = 1). **b.** Individuals with no previous cancer diagnosis that had an autosomal mCA detected (mean number mCAs = 1). **c.** Individuals with a previous cancer diagnosis that had an autosomal mCA detected (mean number mCAs = 1).

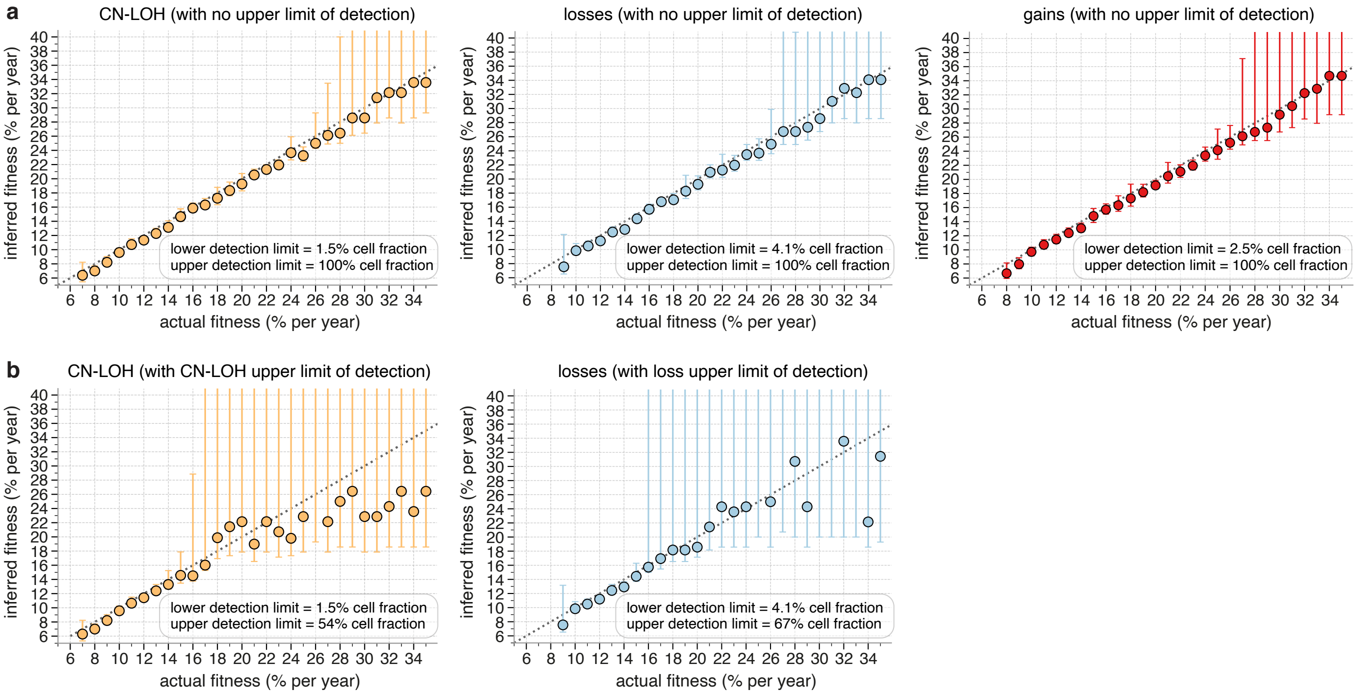


**Supplementary Fig. 5. Effect of upper and lower mCA detection limits on fitness inferences**. (**a**) Actual and inferred fitness effects for simulated data (generated from $n$ = 502,413 'people') with no upper cell fraction limit of detection, but with mCA-class-specific lower cell fraction limits of detection. Error bars show 95% confidence intervals. See Supplementary Note 1 for the number of mCA 'observations' at each simulated fitness effect. (**b**) Actual and inferred fitness effects for simulated data (generated from $n$ = 502,413 'people') with mCA-class-specific upper cell fraction limits of detection and mCA-class-specific lower cell fraction limits of detection. Gains are not shown because their mCA-class-specific upper cell fraction limit of detection is 100%. Error bars show 95% confidence intervals. See Supplementary Table 8 for the number of mCA 'observations' at each simulated fitness effect. For simulated mCAs with >50 'observations', a random sample of 50 'observations' was used for parameter estimation (to reduce compute time).

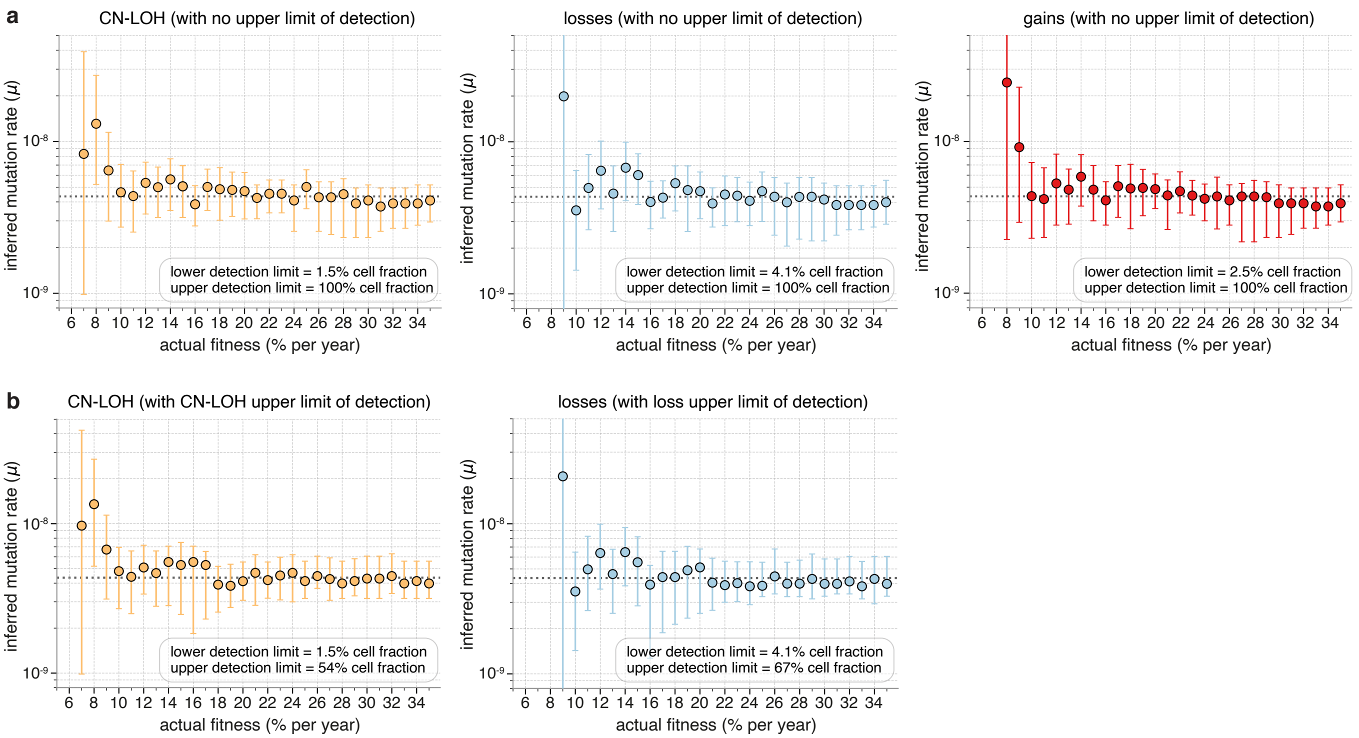


**Supplementary Fig. 6. Effect of upper and lower mCA detection limits on mutation rate inferences, according to mCA fitness effect**. **a.** Inferred mutation rates for simulated data (generated from $n$ = 502,413 'people') with no upper cell fraction limit of detection, but with mCA-class-specific lower cell fraction limits of detection. The actual mutation rate (4.35 $\times 10^{-9}$ per year) is represented by the horizontal dashed line. Error bars show 95% confidence intervals. See Supplementary Note 1 for the number of mCA 'observations' at each simulated fitness effect. **b.** Inferred mutation rates for simulated data (generated from $n$ = 502,413 'people') with mCA-class-specific upper cell fraction limits of detection and mCA-class-specific lower cell fraction limits of detection. The actual mutation rate (4.35 $\times 10^{-9}$ per year) is represented by the horizontal dashed line. Gains are not shown because their mCA-class-specific upper cell fraction limit of detection is 100%. Error bars show 95% confidence intervals. See Supplementary Table 8 for the number of mCA 'observations' at each simulated fitness effect. For simulated mCAs with >50 'observations', a random sample of 50 'observations' was used for parameter estimation (to reduce compute time).

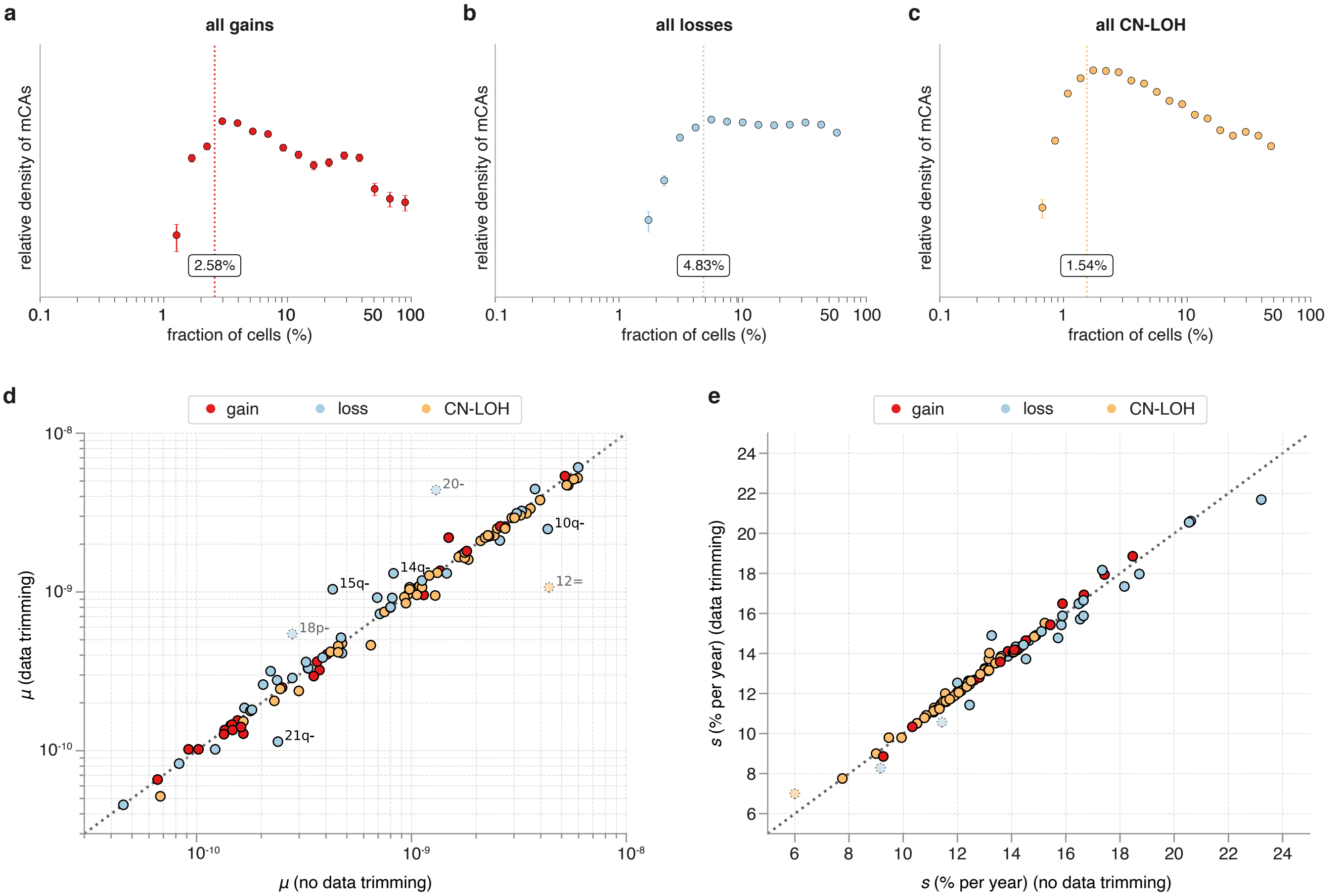


**Supplementary Fig. 7. Effect of false negatives at low cell fraction on fitness and mutation rate inferences**. **a-c.** Cell fraction density histograms for (a) all gains ($n$ = 1410 observations), (b) losses ($n$ = 2248 observations) and (c) CN-LOH events ($n$ = 7433 observations). Vertical dashed lines indicate the cell fraction below which the density of mCAs starts to fall off and thus likely represents the limit of of reliable mCA detection where false negatives become more prevalent. mCAs at cell fractions lower than this cut-off were excluded to assess what effect this had on fitness and mutation rate inferences. Datapoints are presented as mean values +/- SEM. **d-e.** Correlation between mutation rate ($\mu$) (d) and fitness ($s$) inferences (e) with and without data trimming at the cell fractions highlighted in a-c. Faded data points represent mCAs where the total number of data points was <8 after the lower cell fraction mCAs were trimmed (i.e. below the number of datapoints required to form a meaningful cell fraction density histogram).

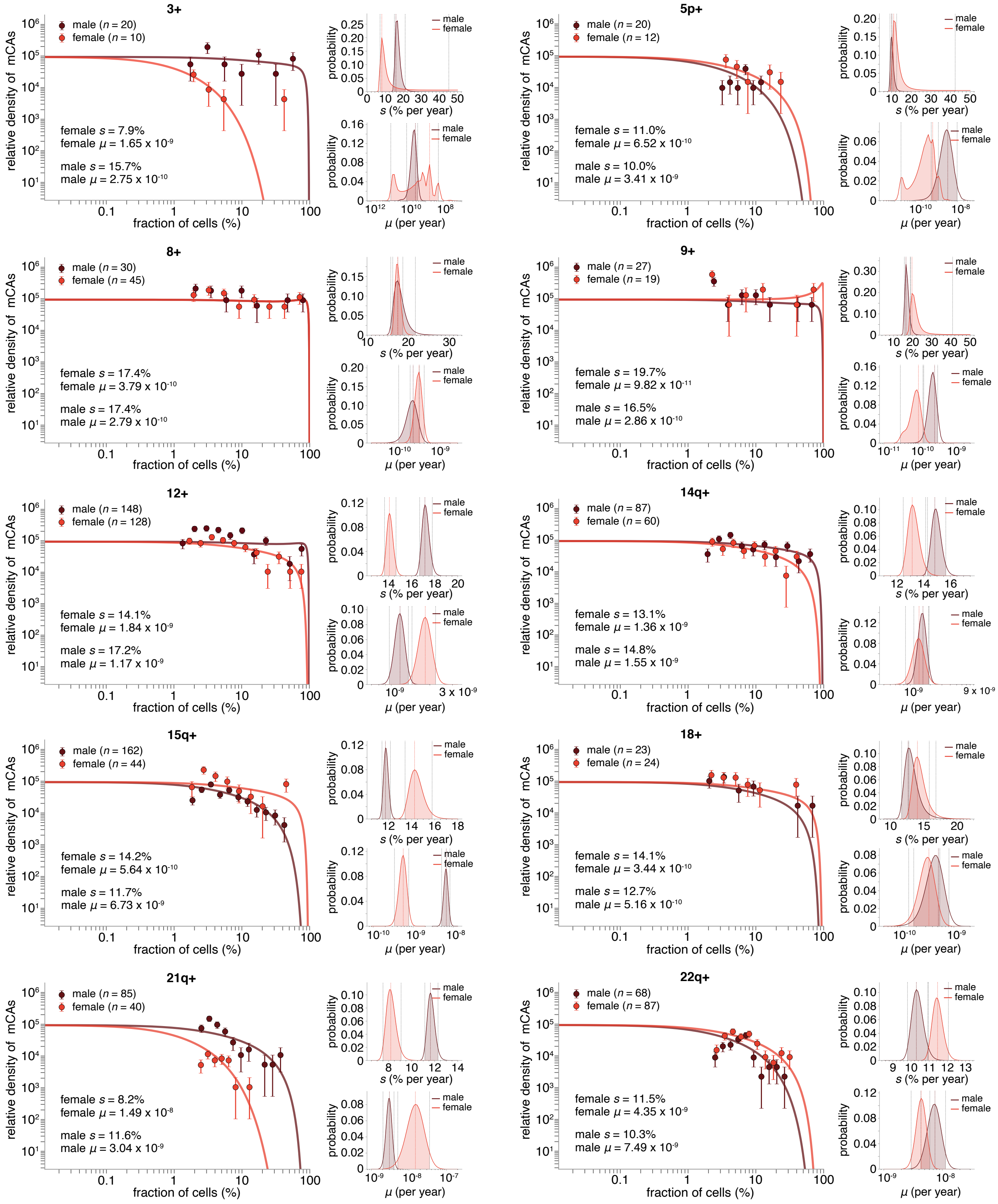


**Supplementary Fig. 8.** **Sex differences in fitness effects and mutation rates: gains**. Only gain events which were observed 10 or more times in men (with a single mCA) and 10 or more times in women (with a single mCA) are shown ($n$ = number of observations of each mCA). Datapoints on the cell fraction density histogram are presented as mean values +/- SEM. Shaded area, between the grey dashed vertical lines on the small subplots, indicates the 95% confidence interval for the estimated fitness effect ($s$) and mutation rate ($\mu$). The coloured vertical dashed line indicates the most likely $s$ and $\mu$ values.

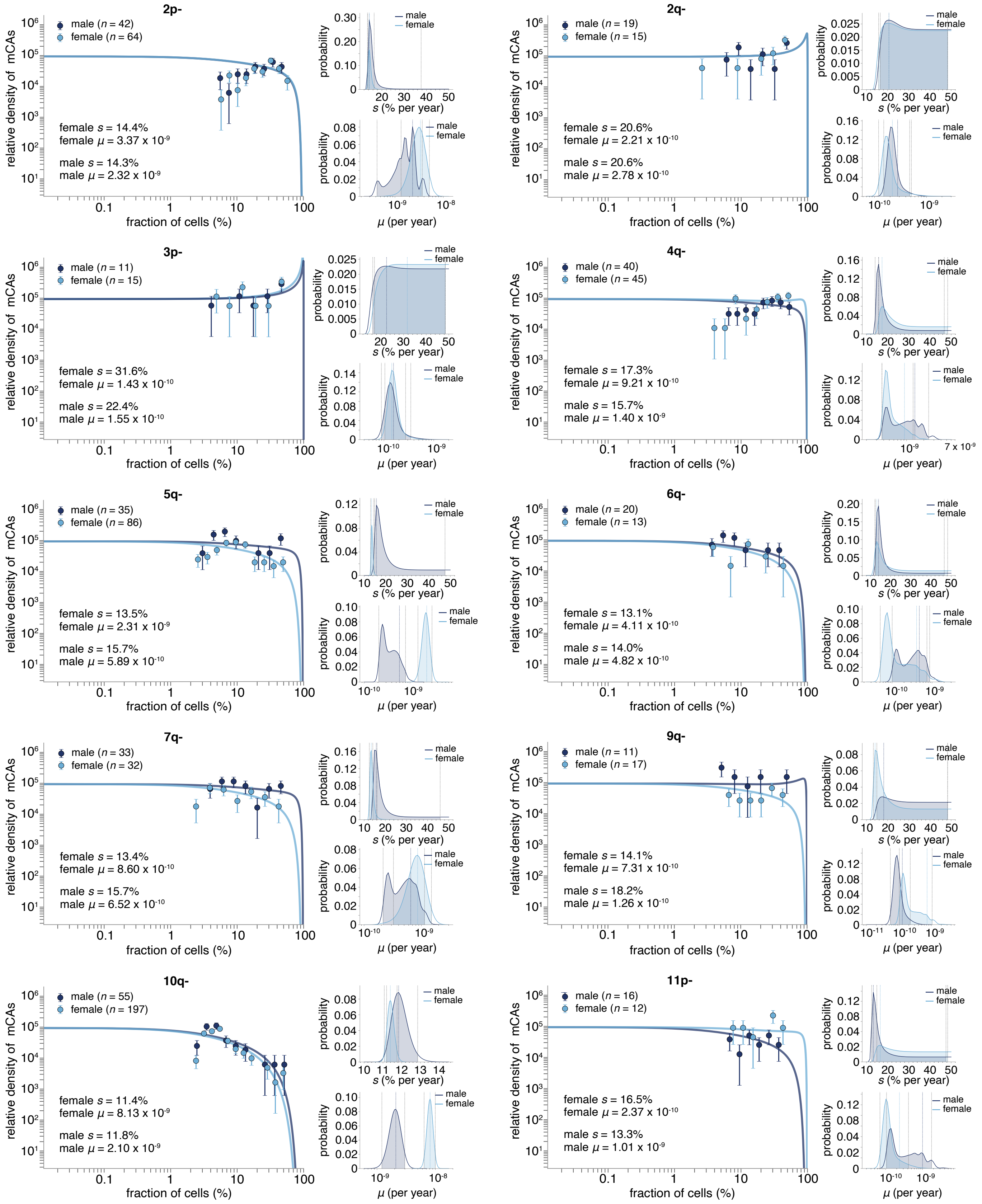


**Supplementary Fig. 9. Sex differences in fitness effects and mutation rates: losses: part 1.** Only loss events which were observed 10 or more times in men (with a single mCA) and 10 or more times in women (with a single mCA) are shown ($n$ = number of observations of each mCA). Datapoints on the cell fraction density histogram are presented as mean values +/- SEM. Shaded area, between the grey dashed vertical lines on the small subplots, indicates the 95% confidence interval for the estimated fitness effect ($s$) and mutation rate ($\mu$). The coloured vertical dashed line indicates the most likely $s$ and $\mu$ values.

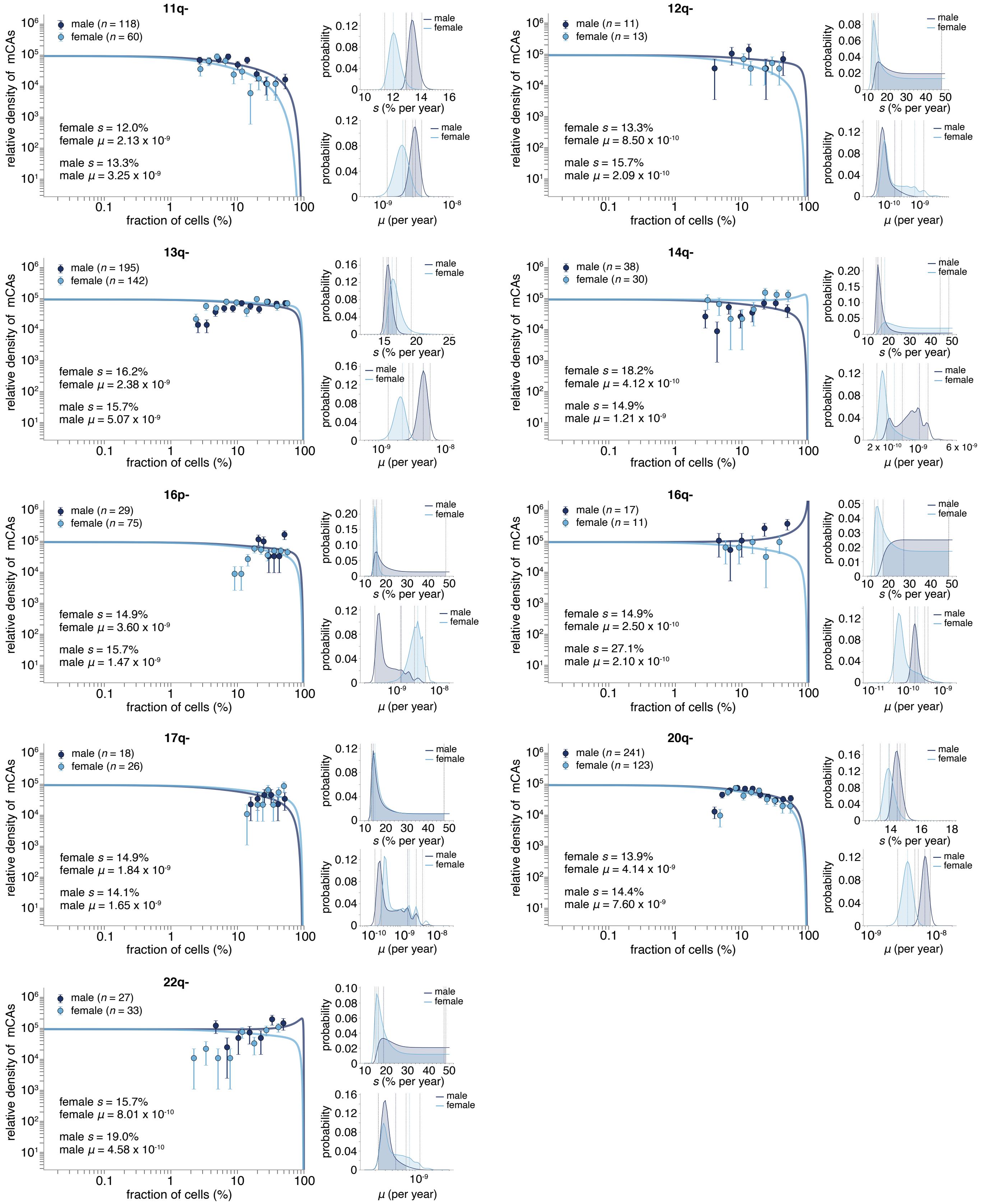


**Supplementary Fig. 10. Sex differences in fitness effects and mutation rates: losses: part 2**. Only loss events which were observed 10 or more times in men (with a single mCA) and 10 or more times in women (with a single mCA) are shown ($n$ = number of observations of each mCA). Datapoints on the cell fraction density histogram are presented as mean values +/- SEM. Shaded area, between the grey dashed vertical lines on the small subplots, indicates the 95% confidence interval for the estimated fitness effect ($s$) and mutation rate ($\mu$). The coloured vertical dashed line indicates the most likely $s$ and $\mu$ values.

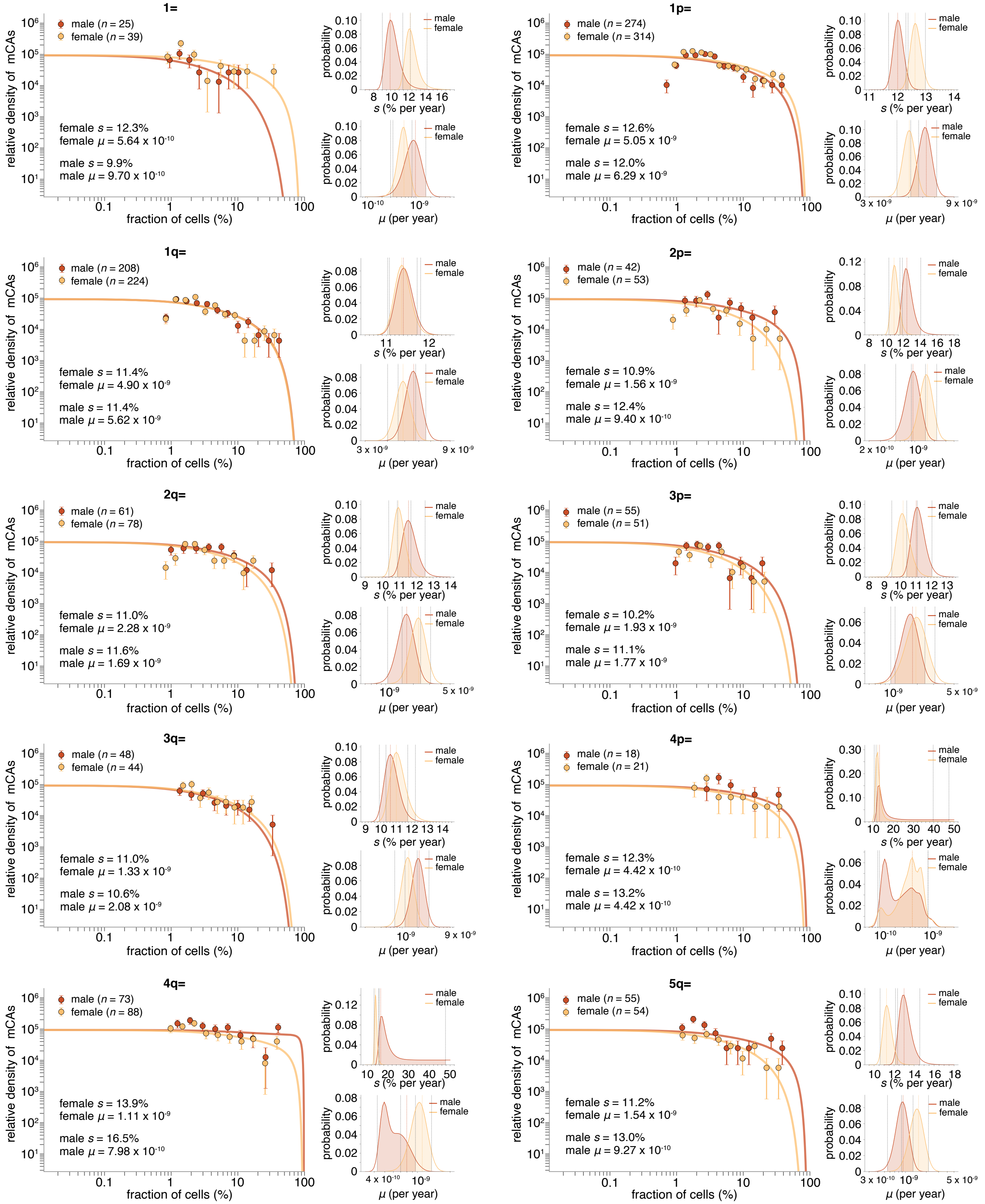


**Supplementary Fig. 11. Sex differences in fitness effects and mutation rates: CNLOH: part 1**. Only CN-LOH events which were observed 10 or more times in men (with a single mCA) and 10 or more times in women (with a single mCA) are shown ($n$ = number of observations of each mCA). Datapoints on the cell fraction density histogram are presented as mean values +/- SEM. Shaded area, between the grey dashed vertical lines on the small subplots, indicates the 95% confidence interval for the estimated fitness effect ($s$) and mutation rate ($\mu$). The coloured vertical dashed line indicates the most likely $s$ and $\mu$ values.

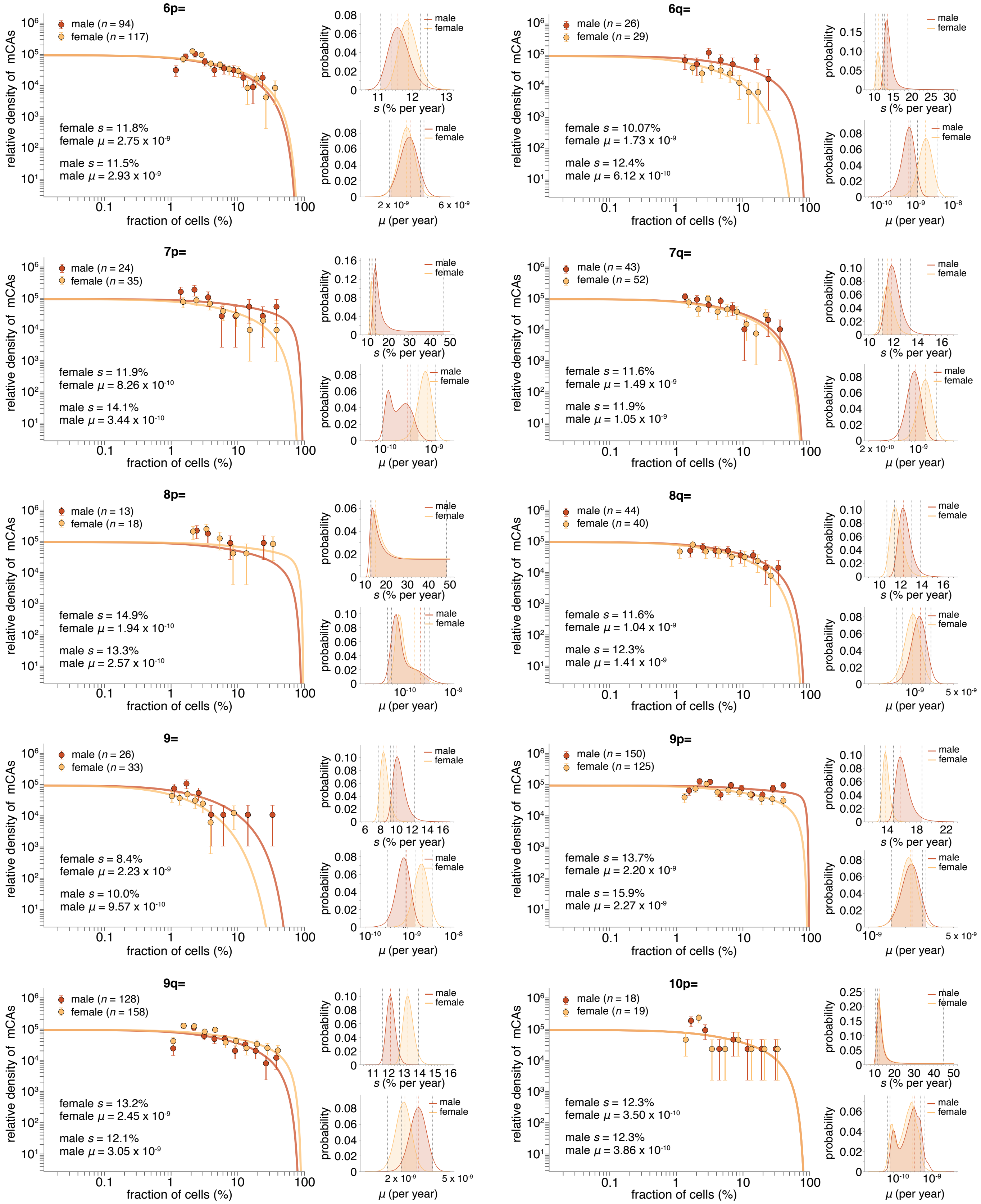


**Supplementary Fig. 12. Sex differences in fitness effects and mutation rates: CN-LOH: part 2**. Only CN-LOH events which were observed 10 or more times in men (with a single mCA) and 10 or more times in women (with a single mCA) are shown ($n$ = number of observations of each mCA). Datapoints on the cell fraction density histogram are presented as mean values +/- SEM. Shaded area, between the grey dashed vertical lines on the small subplots, indicates the 95% confidence interval for the estimated fitness effect ($s$) and mutation rate ($\mu$). The coloured vertical dashed line indicates the most likely $s$ and $\mu$ values.

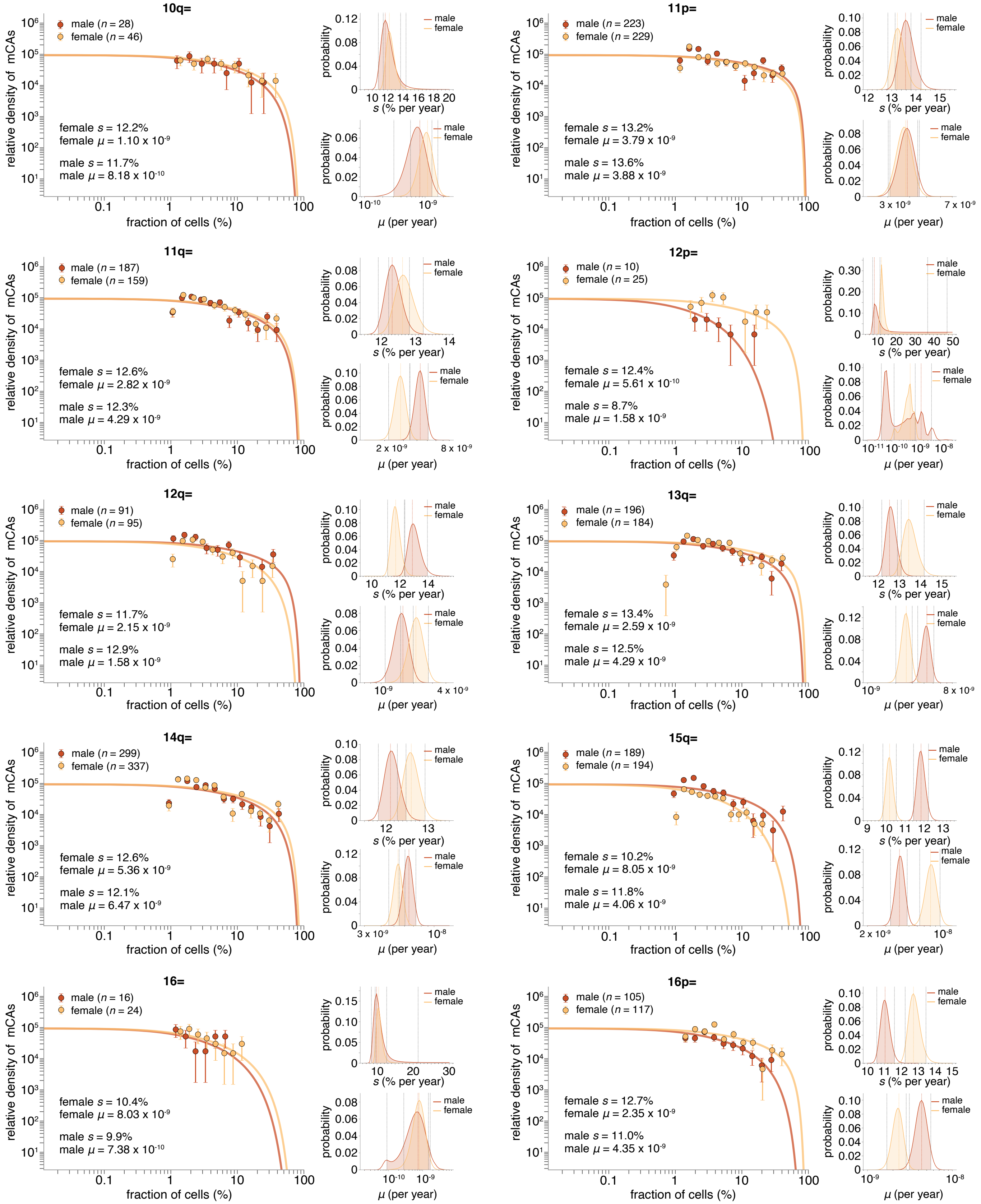


**Supplementary Fig. 13. Sex differences in fitness effects and mutation rates: CN-LOH: part 3**. Only CN-LOH events which were observed 10 or more times in men (with a single mCA) and 10 or more times in women (with a single mCA) are shown ($n$ = number of observations of each mCA). Datapoints on the cell fraction density histogram are presented as mean values +/- SEM. Shaded area, between the grey dashed vertical lines on the small subplots, indicates the 95% confidence interval for the estimated fitness effect ($s$) and mutation rate ($\mu$). The coloured vertical dashed line indicates the most likely $s$ and $\mu$ values.

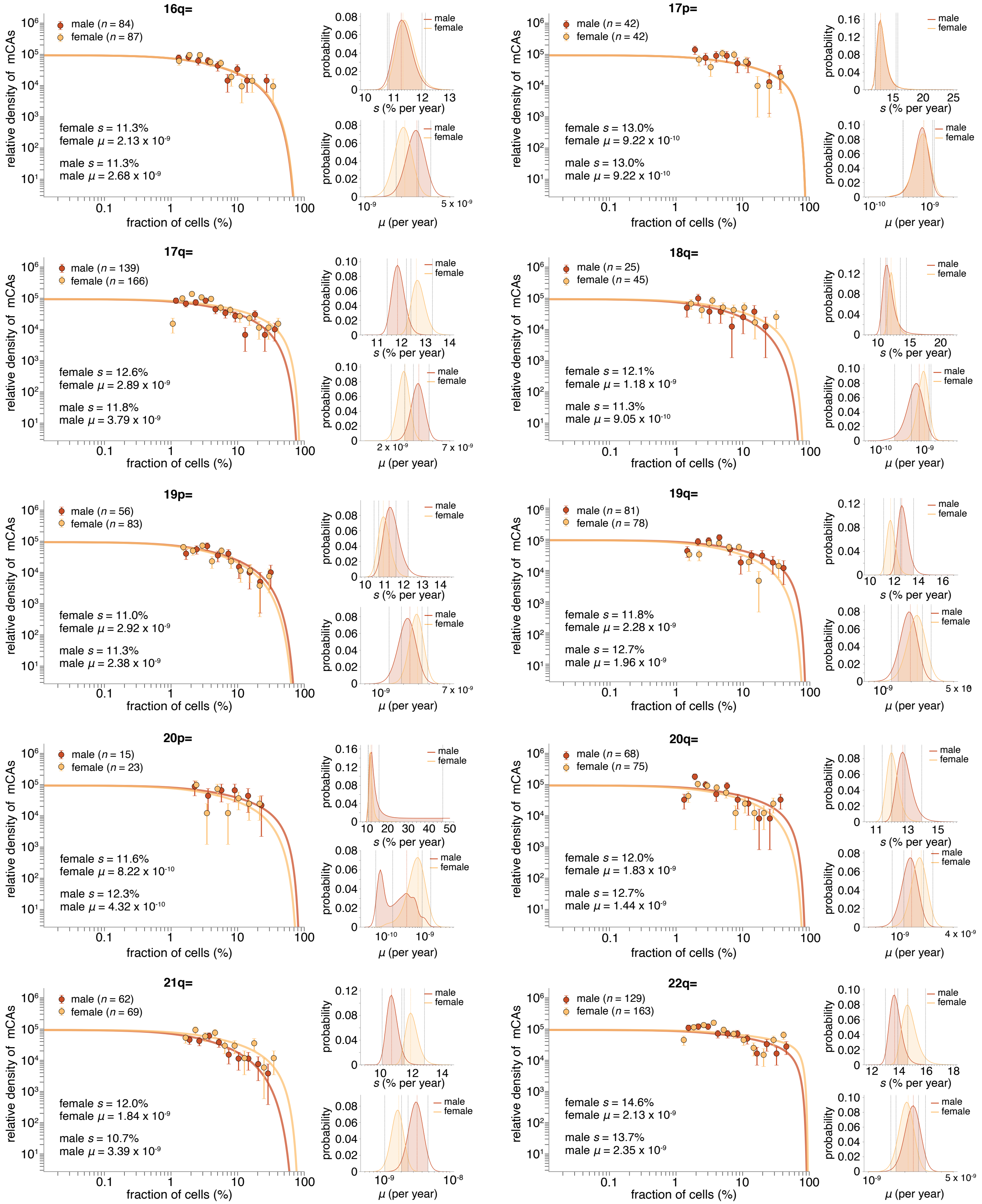


**Supplementary Fig. 14. Sex differences in fitness effects and mutation rates: CN-LOH: part 4.** Only CN-LOH events which were observed 10 or more times in men (with a single mCA) and 10 or more times in women (with a single mCA) are shown ($n$ = number of observations of each mCA). Datapoints on the cell fraction density histogram are presented as mean values +/- SEM. Shaded area, between the grey dashed vertical lines on the small subplots, indicates the 95% confidence interval for the estimated fitness effect ($s$) and mutation rate ($\mu$). The coloured vertical dashed line indicates the most likely $s$ and $\mu$ values.

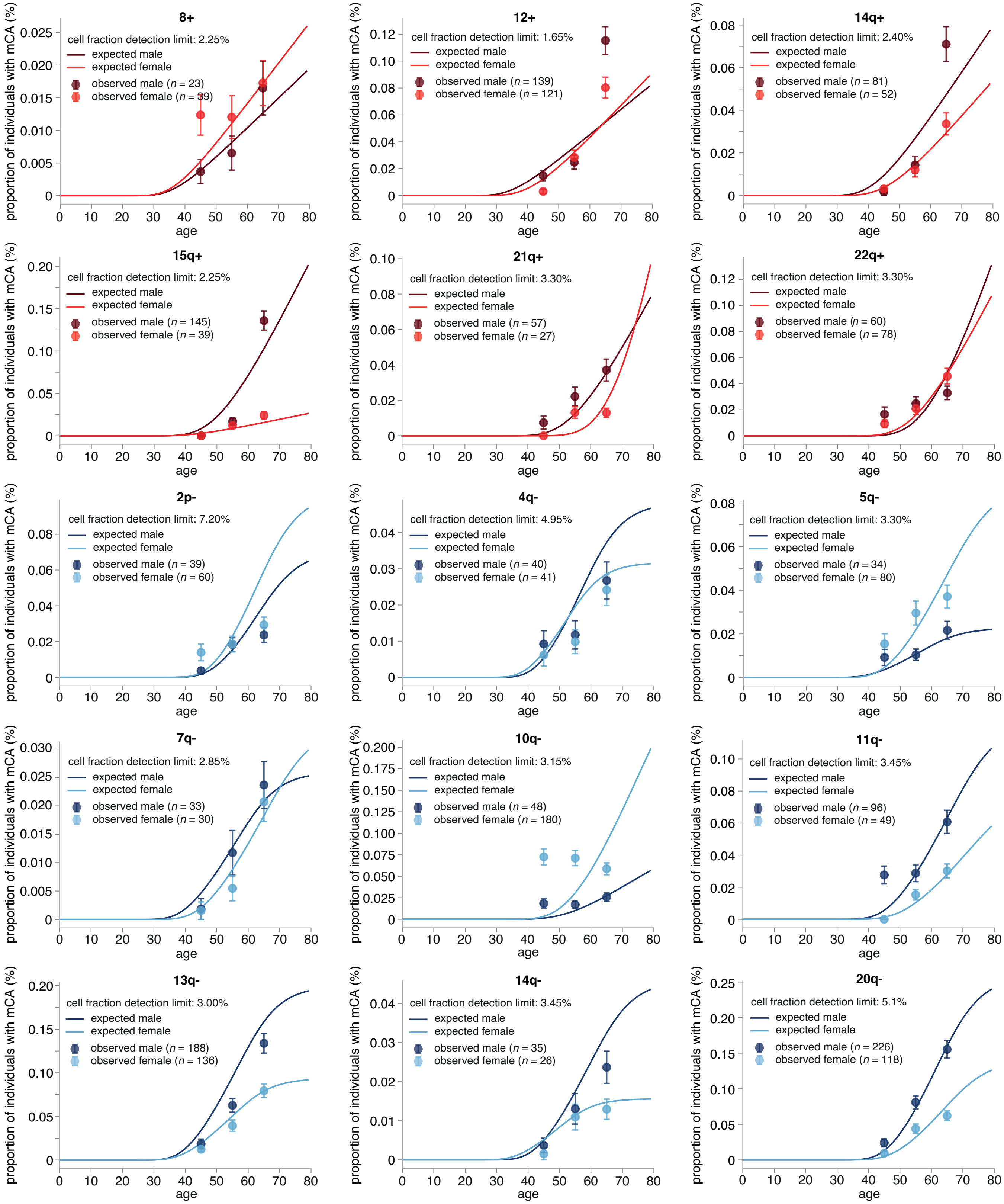


**Supplementary Fig. 15. Predicted age dependence for individual mCAs calculated using sex-specific $\mu$ and $s$ estimates: part 1**. Only mCA events which were observed 30 or more times in both men and women (with a single mCA) are shown. Error bars represent sampling error (+/- 1 SD). The cell fraction limit of detection was taken as the minimum cell fraction observed for the mCA, multiplied by 1.5. *n* = number of observations of each mCA above this cell fraction limit of detection.

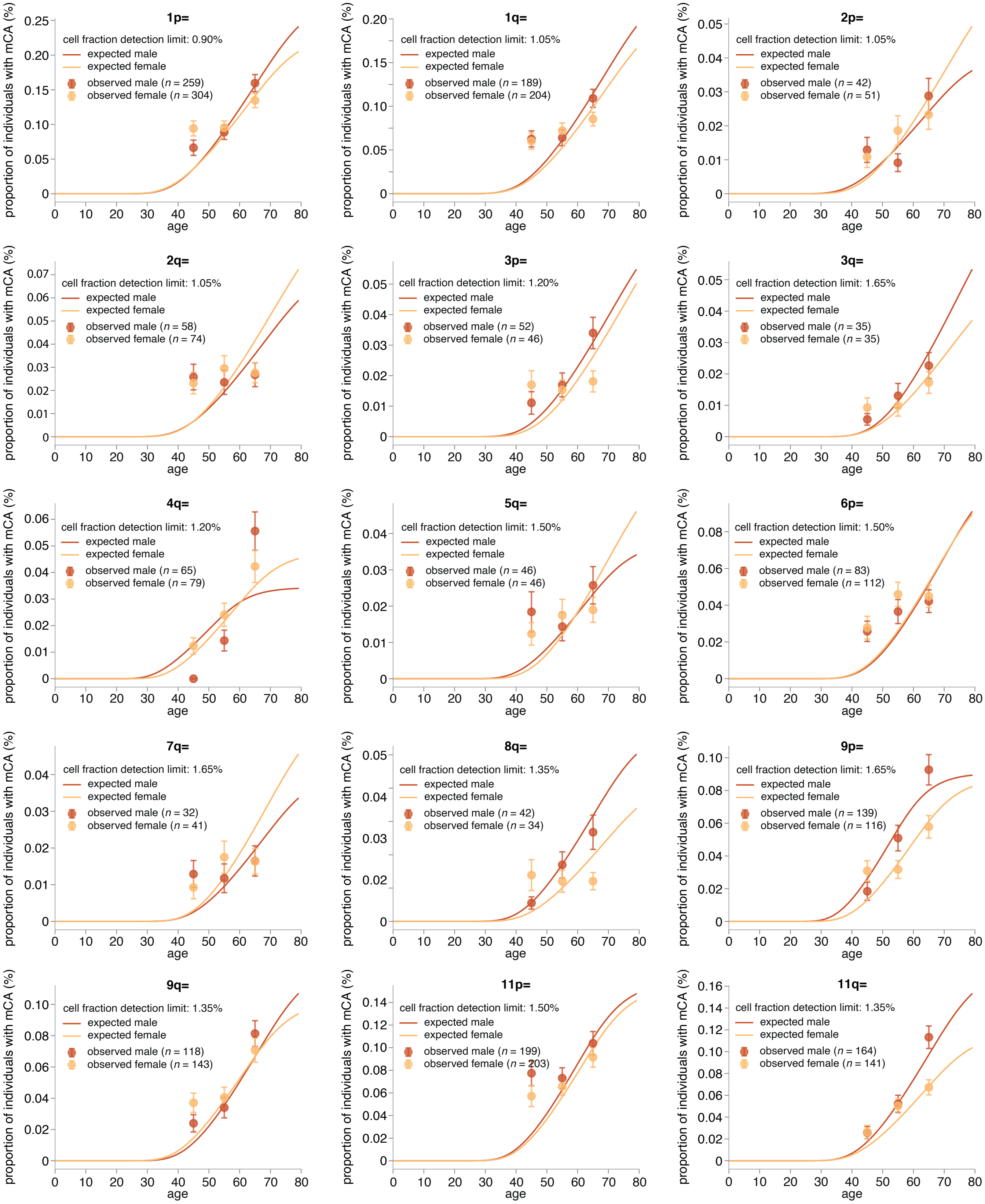


**Supplementary Fig. 16. Predicted age dependence for individual mCAs calculated using sex-specific $\mu$ and $s$ estimates: part 2**. Only mCA events which were observed 30 or more times in both men and women (with a single mCA) are shown. Error bars represent sampling error (+/- 1 SD). The cell fraction limit of detection was taken as the minimum cell fraction observed for the mCA, multiplied by 1.5. $n$ = number of observations of each mCA above this cell fraction limit of detection.

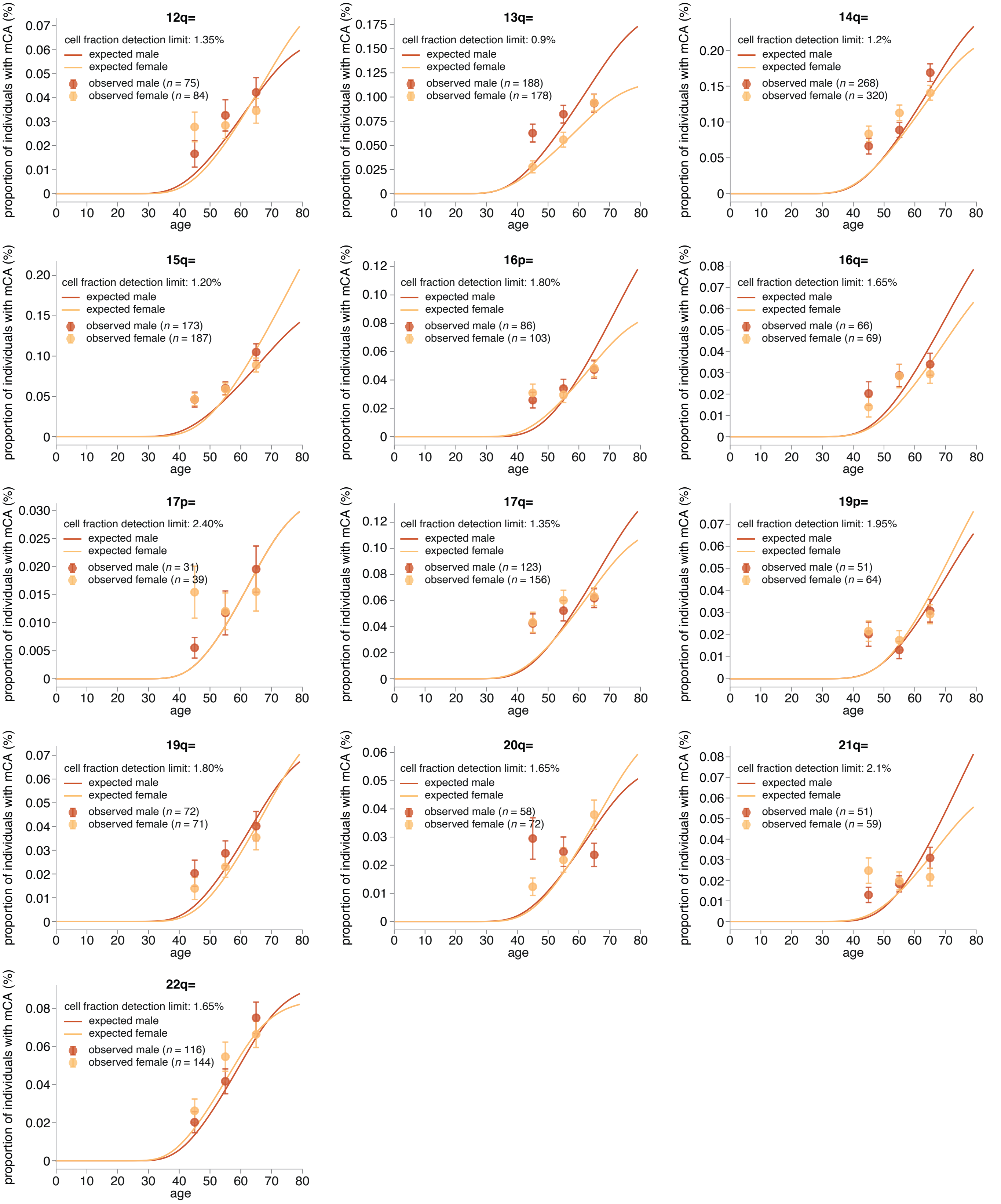


**Supplementary Fig. 17. Predicted age dependence for individual mCAs calculated using sex-specific $\mu$ and $s$ estimates: part 3**. Only mCA events which were observed 30 or more times in both men and women (with a single mCA) are shown. Error bars represent sampling error (+/- 1 SD). The cell fraction limit of detection was taken as the minimum cell fraction observed for the mCA, multiplied by 1.5. *n* = number of observations of each mCA above this cell fraction limit of detection.

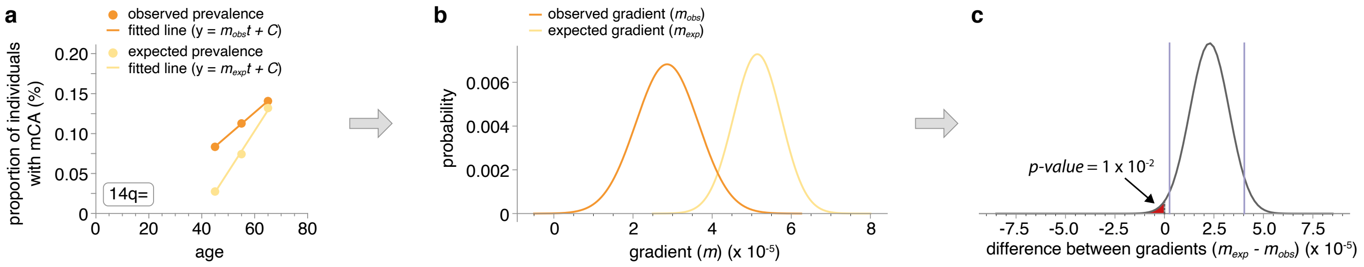


**Supplementary Fig. 18.** **Method for quantifying deviation from expected age dependence.** **a.** A maximum likelihood approach was used to calculate the gradients of the observed ($m_{\text{obs}}$) and expected ($m_{\text{exp}}$) age dependence. Deviation from expected age dependence was calculated as the relative difference between these gradients. **b.** Probability distributions of the gradients for the observed and expected age dependence. **c.** The *p*-value for the difference between the gradients was calculated from the distribution of gradient differences.

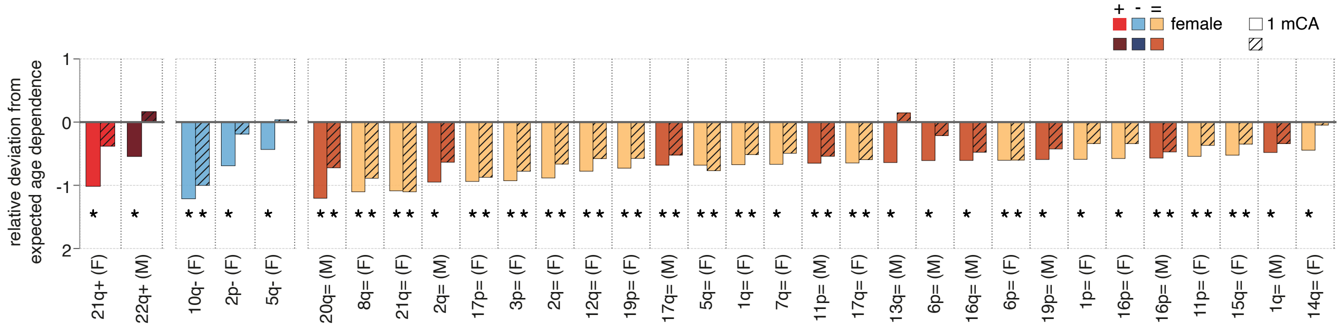


**Supplementary Fig. 19. Deviation from expected age-dependence for mCAs that showed significantly worse age dependence than expected (from Fig. 3), but now including people with multiple mCAs**. The mCAs are ordered, within each class, by largest (left) to smallest (right) deviation from expected age dependence. For each mCA the deviation from expected age-dependence is shown for individuals that had only a single mCA (unhatched bars) and for those that had $\geq$ 1 mCA (hatched bars). Stars indicate whether the deviation was significantly different ($p$ <0.05) from that expected based on the mCA's inferred fitness effect and mutation rate.

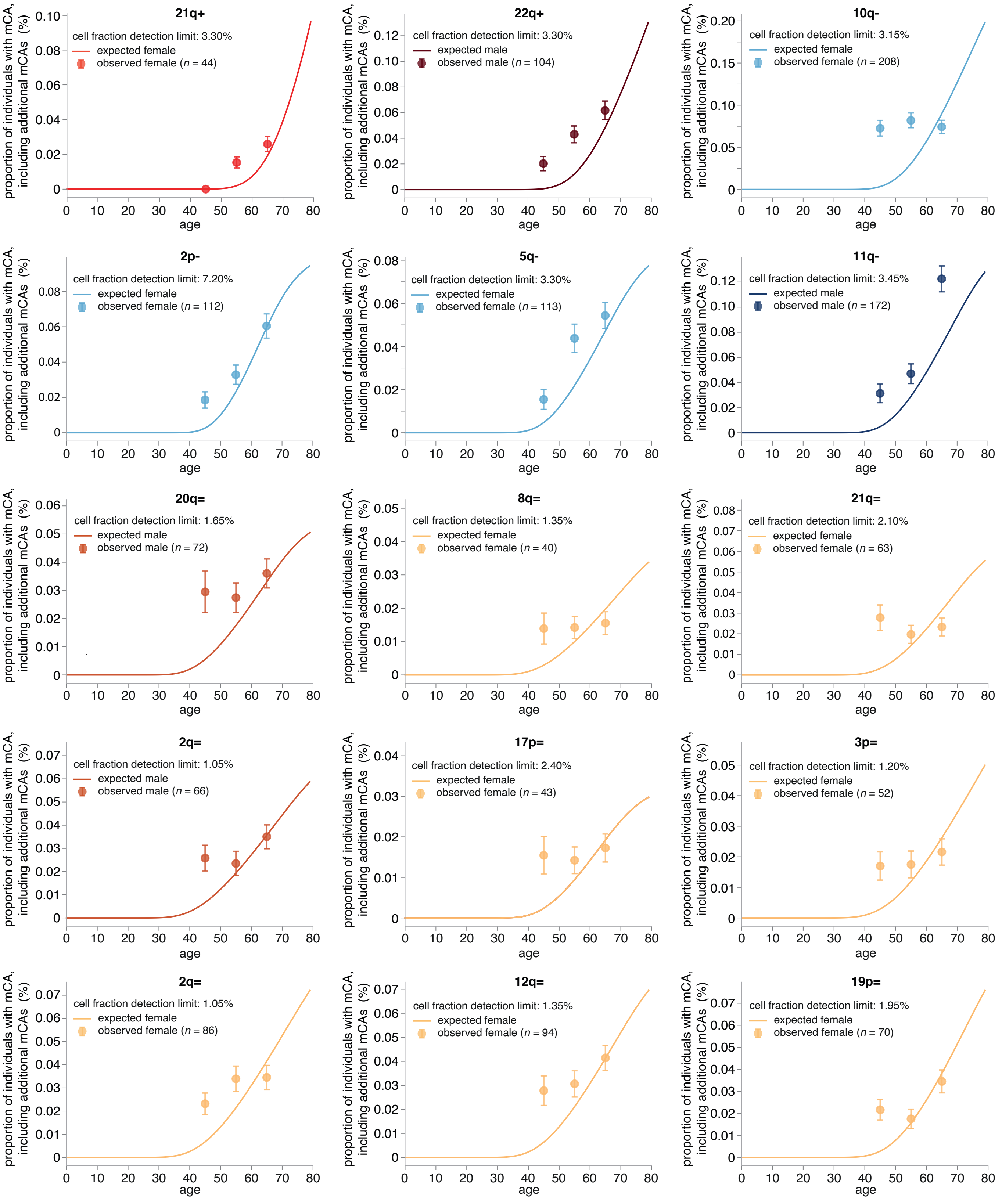


**Supplementary Fig. 20. Age and sex dependence of mCAs with poor age dependence, but including people with multiple mCAs: part 1**. The cell fraction limit of detection used was the minimum cell fraction observed for the mCA, multiplied by 1.5. $n$ = number of observations of each mCA above this cell fraction limit of detection. The 'expected prevalence' is for 'at least 1' mCA, using the mCA-specific fitness effect and mutation rate. Error bars represent sampling error (+/- 1 SD).

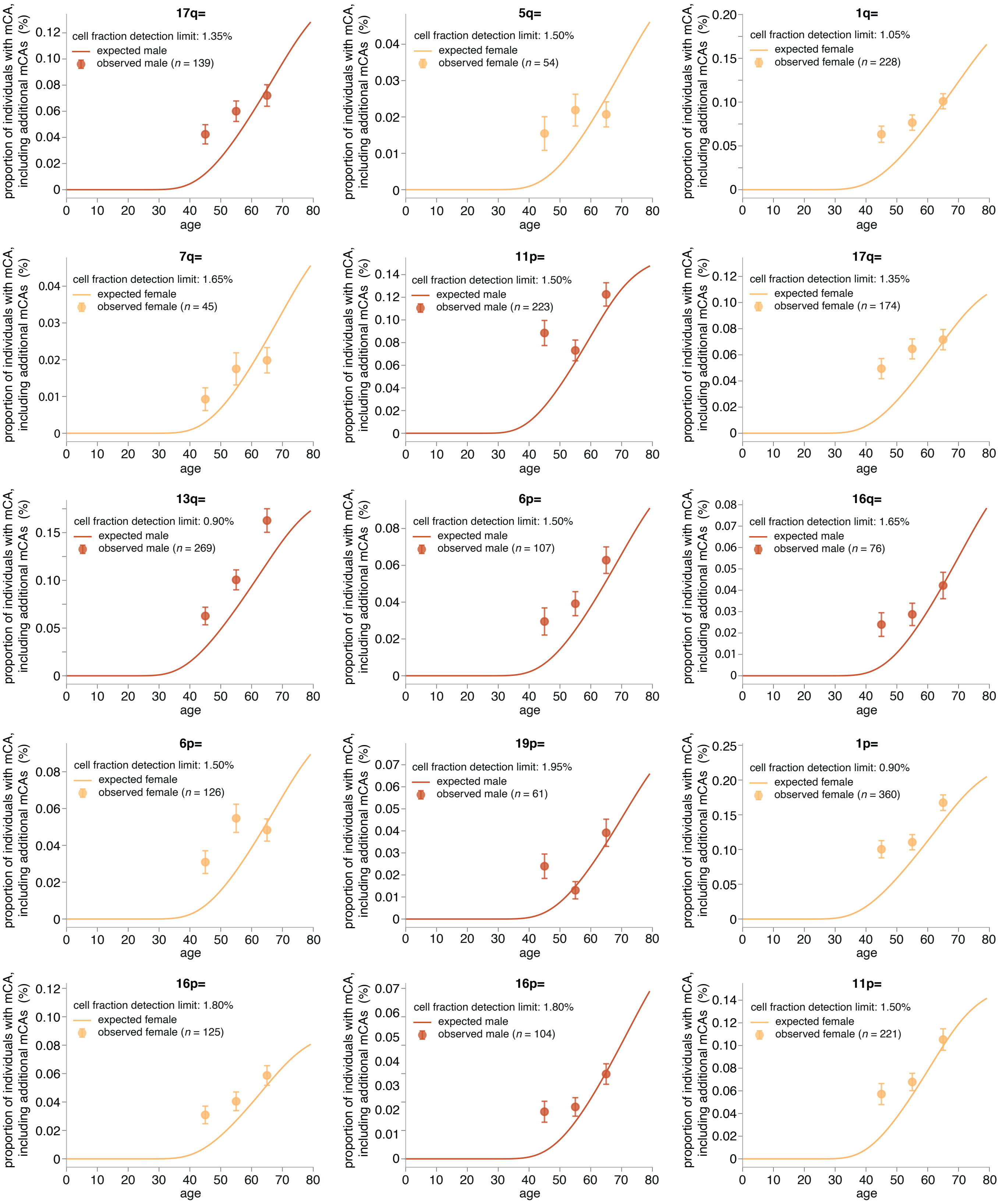


**Supplementary Fig. 21. Age and sex dependence of mCAs with poor age dependence, but including people with multiple mCAs: part 2**. The cell fraction limit of detection used was the minimum cell fraction observed for the mCA, multiplied by 1.5. *n* = number of observations of each mCA above this cell fraction limit of detection. The 'expected prevalence' is for 'at least 1' mCA, using the mCA-specific fitness effect and mutation rate. Error bars represent sampling error (+/- 1 SD).

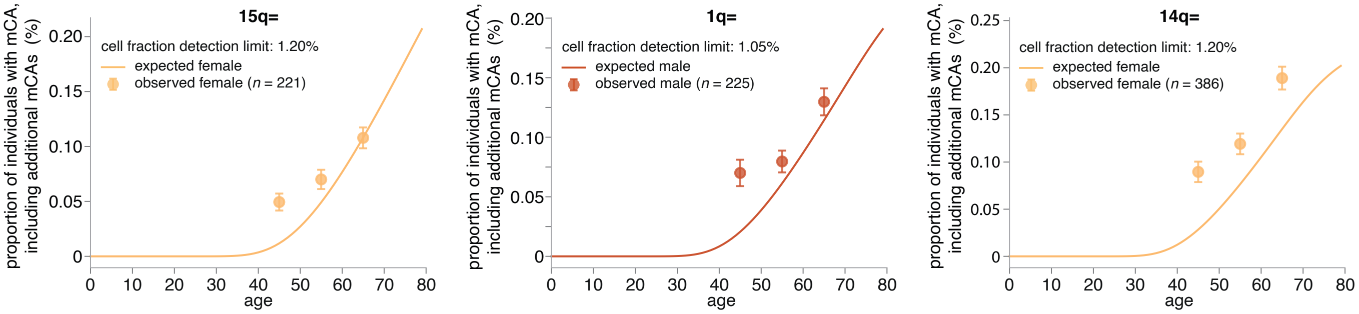


**Supplementary Fig. 22. Age and sex dependence of mCAs with poor age dependence, but including people with multiple mCAs: part 3**. The cell fraction limit of detection used was the minimum cell fraction observed for the mCA, multiplied by 1.5. *n* = number of observations of each mCA above this cell fraction limit of detection. The 'expected prevalence' is for 'at least 1' mCA, using the mCA-specific fitness effect and mutation rate. Error bars represent sampling error (+/- 1 SD).

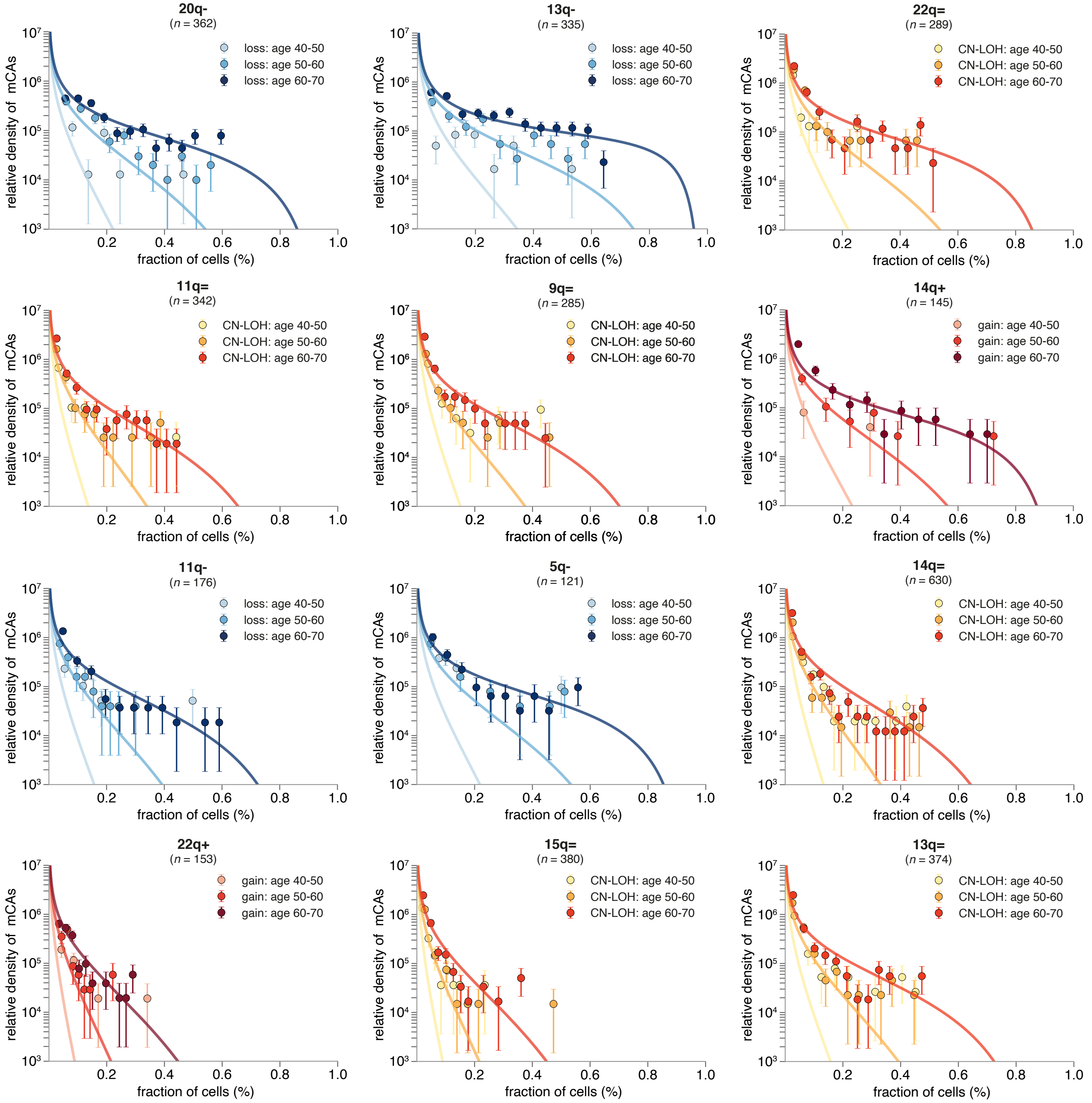


**Supplementary Fig. 23. Age dependence of the distribution of clone sizes for specific mCAs**. The density of cell-fractions estimates for the 12 mCAs with >100 datapoints that showed the best overall age prevalence (as determined in Fig. 3) are shown. For these mCAs we plotted the observed density of cell fractions (datapoints) for the 3 different age groups and compared this to the density predicted by our framework (solid lines). *n* = number of observations of each mCA across the 3 different age groups. Datapoints are presented as mean values +/- SEM.

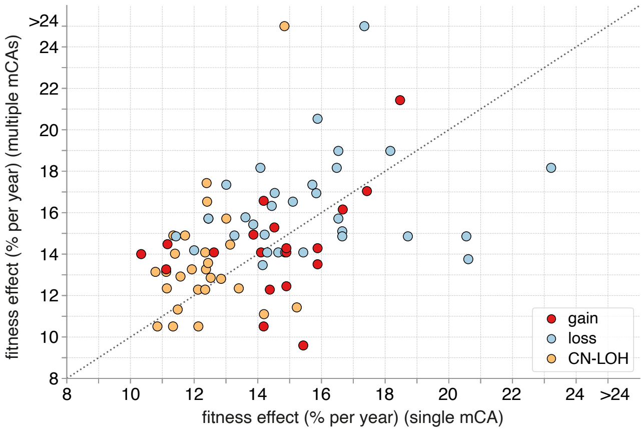


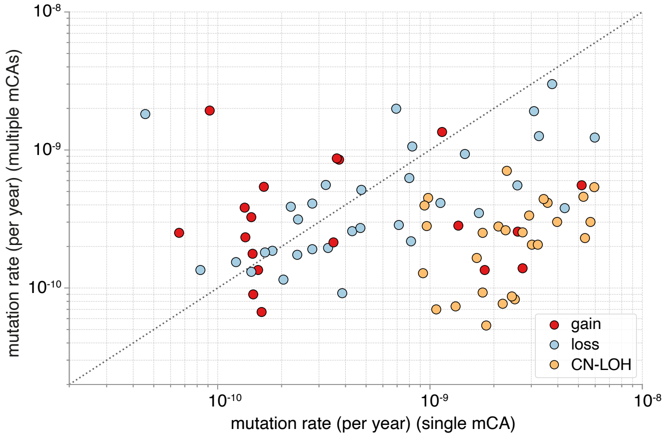


**Supplementary Fig. 24. Fitness effects and mutation rates for mCAs observed as single events vs. observed with additional mCAs**. Only mCAs that were observed in 8 or more individuals that also had additional mCAs are shown.

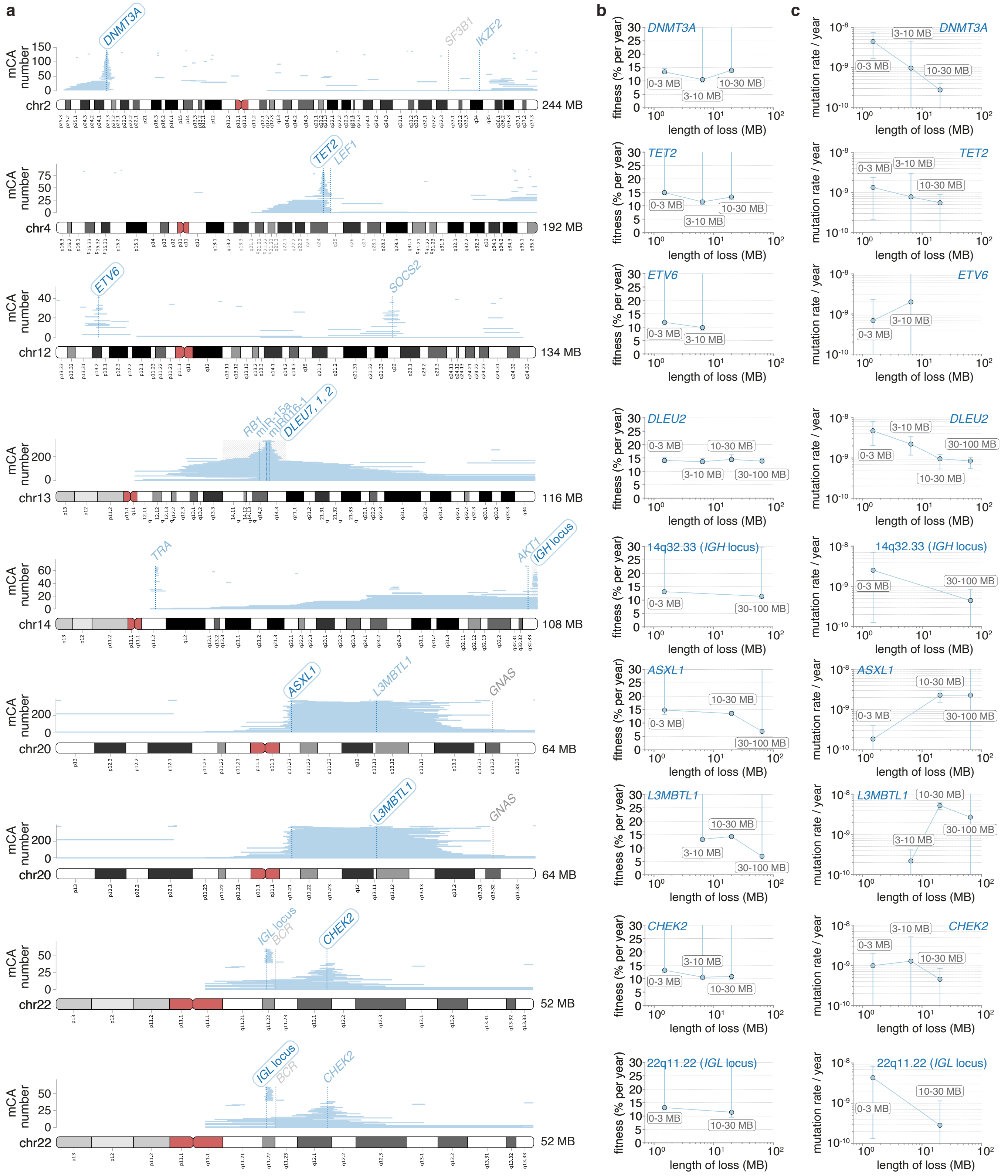


**Supplementary Fig. 25. Length dependence of fitness effects and mutation rates for loss events. a.** Strong clustering of loss events involving genes commonly mutated in clonal haematopoiesis and haematological malignancies was observed. **b.** Fitness effects were calculated for all losses that involved the particular gene highlighted in (a), separated into broad length categories. Error bars represent 95% confidence intervals. **c.** Mutation rates were calculated for all losses that involved the particular gene highlighted in (a), separated into broad length categories. Error bars represent 95% confidence intervals.

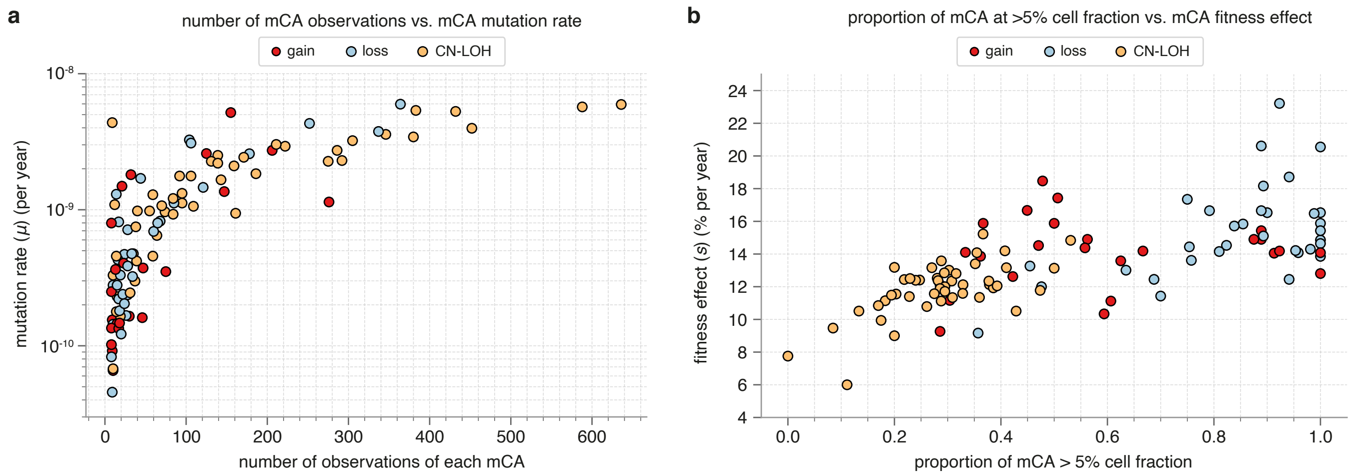


**Supplementary Fig. 26. a. Correlation between number of mCA observations and mCA mutation rate.** mCAs with similar mutation rates can have very different prevalence, e.g. some mCAs with mutation rates of $1 \times 10^{-9}$ per year were observed <10 times, whereas others with a similar mutation rate were observed >150 times. This is because the mCA prevalence is determined by both the mutation rate and the fitness effect, with higher fitness effects resulting in more mCAs expanding to detectable levels. **b. Correlation between proportion of mCA events at cell fraction >5% and mCA fitness effect.** Whilst mCAs with higher fitness effects are generally more likely to be found at higher cell fraction, mCAs observed in a similar proportion of people at >5% cell fraction can have very different fitness effects, e.g. some mCAs in which >80% were found at >5% cell fraction had fitness effects of $\sim$12% per year, whereas others had fitness effects of >28% per year.

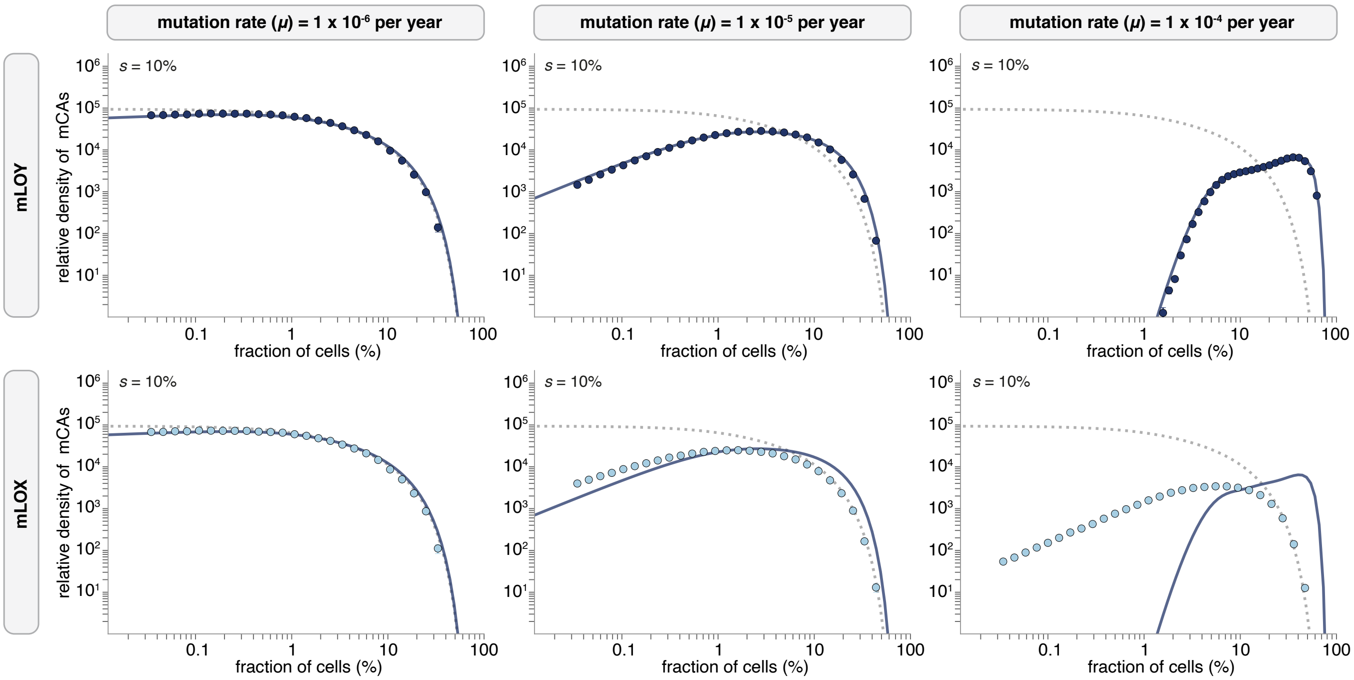


**Supplementary Fig. 27.** **Distribution of simulated mLOY and mLOX cell fractions, with high mutation rates.** Top row shows distribution of simulated mLOY events (in which independent mLOY events would not be distinguishable in an individual) as a function of increasing mutation rate (left to right). Datapoints are results of stochastic simulations (see Supplementary Note 1). Grey dashed line shows the theoretical expectation assuming independent mCA events are distinguishable. Dark blue line shows the analytical expression for the distribution from eq. 8 where independent events are not distinguishable and therefore their 'cell fraction' is a sum of the independent cell fractions in that individual. Bottom row shows distribution of simulated mLOX events as a function of increasing mutation rate (left to right). In this case, the measured 'cell fraction' is the absolute difference between the sum of the independent mLOX events affecting the maternal X-chromosome and the sum of the independent mLOY events affecting the paternal X-chromosome. Grey dashed and dark blue lines are as described for the top row.

## Supplement References

1 Loh, P.-R., Genovese, G. & McCarroll, S. A. Monogenic and polygenic inheritance become instruments for clonal selection. *Nature* **584**, 136–141 (2020).
2 Watson, C. J. et al. The evolutionary dynamics and fitness landscape of clonal hematopoiesis. *Science* **367**, 1449–1454 (2020).
3 Levy, S. F. et al. Quantitative evolutionary dynamics using high-resolution lineage tracking. *Nature* **519**, 181–186 (2015).
