# Supplementary material for: Mutation rates and fitness consequences of mosaic chromosomal alterations in blood
Source: Nat Genet. 2023 Sep 11;55(10):1677–85. doi: 10.1038/s41588-023-01490-z (PMC10562253; doi:10.1038/s41588-023-01490-z)
Supplement: Supplementary file 2 — Reporting Summary [file 41588_2023_1490_MOESM2_ESM.pdf]

nature portfolio

Corresponding author(s): Jamie Blundell, Caroline Watson

Last updated by author(s): Apr 20, 2026

Reporting Summary

Nature Portfolio wishes to improve the reproducibility of the work that we publish. This form provides structure for consistency and transparency in reporting. For further information on Nature Portfolio policies, see our Editorial Policies and the Editorial Policy Checklist.

Statistics

For all statistical analyses, confirm that the following items are present in the figure legend, table legend, main text, or Methods section.

n/a	Confirmed	
☐	☒	The exact sample size (*n*) for each experimental group/condition, given as a discrete number and unit of measurement
☒	☐	A statement on whether measurements were taken from distinct samples or whether the same sample was measured repeatedly
☐	☒	The statistical test(s) used AND whether they are one- or two-sided<br>*Only common tests should be described solely by name; describe more complex techniques in the Methods section.*
☒	☐	A description of all covariates tested
☒	☐	A description of any assumptions or corrections, such as tests of normality and adjustment for multiple comparisons
☐	☒	A full description of the statistical parameters including central tendency (e.g. means) or other basic estimates (e.g. regression coefficient) AND variation (e.g. standard deviation) or associated estimates of uncertainty (e.g. confidence intervals)
☐	☒	For null hypothesis testing, the test statistic (e.g. *F*, *t*, *r*) with confidence intervals, effect sizes, degrees of freedom and *P* value noted<br>*Give P values as exact values whenever suitable.*
☒	☐	For Bayesian analysis, information on the choice of priors and Markov chain Monte Carlo settings
☒	☐	For hierarchical and complex designs, identification of the appropriate level for tests and full reporting of outcomes
☐	☒	Estimates of effect sizes (e.g. Cohen's *d*, Pearson's *r*), indicating how they were calculated

Our web collection on statistics for biologists contains articles on many of the points above.

Software and code

Policy information about availability of computer code

Data collection	No new data was collected.
Data analysis	Custom code used for data analysis is available on Blundell Lab GitHub page: https://github.com/the-blundell-lab/mCA-mutation-rates-fitness-consequences (DOI: 10.5281/zenodo.19662539) . The following publicly available software packages were used in analysis and Figure generation: Jupyter notebook 6.4.5, Python 3.7.4, Pandas 1.3.4, NumPy 1.20.1, Matplotlib 3.4.3, Scipy 1.7.1, csv 1.0, seaborn 0.11.2.

For manuscripts utilizing custom algorithms or software that are central to the research but not yet described in published literature, software must be made available to editors and reviewers. We strongly encourage code deposition in a community repository (e.g. GitHub). See the Nature Portfolio guidelines for submitting code & software for further information.

Data

Policy information about availability of data

All manuscripts must include a data availability statement. This statement should provide the following information, where applicable:

- Accession codes, unique identifiers, or web links for publicly available datasets
- A description of any restrictions on data availability
- For clinical datasets or third party data, please ensure that the statement adheres to our policy

The mCA calls used in our analysis are available from UK Biobank (Return 3094), via an application process described at http://www.ukbiobank.ac.uk/using-the-

resource/. Autosomal mCA calls are also available from Loh, P-R., Genovese G. & McCarroll S. Monogenic and polygenic inheritance become instruments for clonal selection, Supplementary Data: https://doi.org/10.1038/s41586-020-2430-6 (2020).

Human research participants

Policy information about studies involving human research participants and Sex and Gender in Research.

Reporting on sex and gender	Where enough data was available, we calculated sex-specific fitness effects and mutation rates for mCAs (Figure 2c and Supplementary Note 3). Sex was defined as reported by UK Biobank.
Population characteristics	The mCA calls were generated by Loh et al (doi: 10.1038/s41586-020-2430-6) using SNP array data from 482,789 UK Biobank participants, out of a total 502,650 UK Biobank participants who had the following characteristics: Average age 56.52 years (range 37-73 years); Sex: 54.4% female, 45.6% male; Ethnicity: 94% White British, 2% Asian or Asian British, 1.6% Black or Black British, 0.6% Mixed, 0.3% Chinese, 0.9% Other.
Recruitment	No new study participants were recruited for this research.
Ethics oversight	The North West Multi-centre Research Ethics Committee (MREC) reviewed and approved the UK Biobank scientific protocol and operational procedures (REC reference number: 21/NW/0157) and all participants provided signed informed consent at enrolment.

Note that full information on the approval of the study protocol must also be provided in the manuscript.

Field-specific reporting

Please select the one below that is the best fit for your research. If you are not sure, read the appropriate sections before making your selection.

☒ Life sciences ☐ Behavioural & social sciences ☐ Ecological, evolutionary & environmental sciences

For a reference copy of the document with all sections, see nature.com/documents/nr-reporting-summary-flat.pdf

Life sciences study design

All studies must disclose on these points even when the disclosure is negative.

Sample size	No statistical methods were used to predetermine sample size. The mCA calls used in our analysis (Loh et al 2020 (doi: 10.1038/s41586-020-2430-6) and UK Biobank Return 3094) were generated by analysis of all available UK Biobank samples.
Data exclusions	When calling mCAs, Loh et al excluded individuals with low genotyping quality (B-allele frequency s.d. >0.11 at heterozygous sites), individuals with evidence of possible sample contamination, and individuals who had withdrawn consent. For the mLOY calls (from UK Biobank Return 3094), which were based on BAF and LRR measurements in PAR1, we only included those >2MB in size, to reduce the risk of including focal loss events on the Y chromosome. For the mLOX calls (from UK Biobank Return 3094) we only included those >125MB in size, to reduce the risk of including focal loss events on the X chromosome. For fitness effect and mutation rate parameter estimation, mLOX, mLOY and simulated mCA calls were each downsampled to 50 calls (using NumPy 1.20.1 numpy.random.sample with numpy.random.seed(seed = 3, version =2) to reduce compute time.
Replication	Not applicable - experimental replication was not attempted in the generation of the mCA calls used in our analysis.
Randomization	For fitness effect and mutation rate parameter estimation, mLOX, mLOY and simulated mCA calls were each downsampled to 50 calls (using NumPy 1.20.1 numpy.random.sample with numpy.random.seed(seed = 3, version =2) to reduce compute time.
Blinding	Blinding was not performed for this study as the identify of the mCA call was essential for our analysis.

Reporting for specific materials, systems and methods

We require information from authors about some types of materials, experimental systems and methods used in many studies. Here, indicate whether each material, system or method listed is relevant to your study. If you are not sure if a list item applies to your research, read the appropriate section before selecting a response.

Materials & experimental systems

n/a	Involved in the study	
☒	☐	Antibodies
☒	☐	Eukaryotic cell lines
☒	☐	Palaeontology and archaeology
☒	☐	Animals and other organisms
☒	☐	Clinical data
☒	☐	Dual use research of concern

Methods

n/a	Involved in the study	
☒	☐	ChIP-seq
☒	☐	Flow cytometry
☒	☐	MRI-based neuroimaging
